# Multi-omics identification of GPCR gene features in lung adenocarcinoma based on multiple machine learning combinations

**DOI:** 10.7150/jca.90990

**Published:** 2024-01-01

**Authors:** Yiluo Xie, Xinyu Pan, Ziqiang Wang, Hongyu Ma, Wanjie Xu, Hua Huang, Jing Zhang, Xiaojing Wang, Chaoqun Lian

**Affiliations:** 1 Department of Clinical Medicine, Bengbu Medical College, Bengbu 233030, China.; 2 Department of Medical Imaging, Bengbu Medical College, Bengbu 233030, China.; 3 Research Center of Clinical Laboratory Science, Bengbu Medical College, Bengbu 233030, China.; 4 Department of Genetics, School of Life Sciences, Bengbu Medical College, Bengbu 233000, China.; 5 Anhui Province Key Laboratory of Clinical and Preclinical Research in Respiratory Disease, Molecular Diagnosis Center pulmonary critical care medicine, First Affiliated Hospital of Bengbu Medical College, Bengbu, 233000, China.

**Keywords:** Lung adenocarcinoma, G-protein-coupled receptors, Multi-omics, Single-cell RNA-seq, Prognosis, Immunotherapy efficacy, Machine learning

## Abstract

**Background:** Lung adenocarcinoma is a common malignant tumor that ranks second in the world and has a high mortality rate. G protein-coupled receptors (GPCRs) have been reported to play an important role in cancer; however, G protein-coupled receptor-associated features have not been adequately investigated.

**Methods:** In this study, GPCR-related genes were screened at single-cell and bulk transcriptome levels based on AUcell, single-sample gene set enrichment analysis (ssGSEA) and weighted gene co-expression network (WGCNA) analysis. And a new machine learning framework containing 10 machine learning algorithms and their multiple combinations was used to construct a consensus G protein-coupled receptor-related signature (GPCRRS). GPCRRS was validated in the training set and external validation set. We constructed GPCRRS-integrated nomogram clinical prognosis prediction tools. Multi-omics analyses included genomics, single-cell transcriptomics, and bulk transcriptomics to gain a more comprehensive understanding of prognostic features. We assessed the response of risk subgroups to immunotherapy and screened for personalized drugs targeting specific risk subgroups. Finally, the expression of key GPCRRS genes was verified by RT-qPCR.

**Results:** In this study, we identified 10 GPCR-associated genes that were significantly associated with the prognosis of lung adenocarcinoma by single-cell transcriptome and bulk transcriptome. Univariate and multivariate showed that the survival rate was higher in low risk than in high risk, which also suggested that the model was an independent prognostic factor for LUAD. In addition, we observed significant differences in biological function, mutational landscape, and immune cell infiltration in the tumor microenvironment between high and low risk groups. Notably, immunotherapy was also relevant in the high and low risk groups. In addition, potential drugs targeting specific risk subgroups were identified.

**Conclusion:** In this study, we constructed and validated a lung adenocarcinoma G protein-coupled receptor-related signature, which has an important role in predicting the prognosis of lung adenocarcinoma and the effect of immunotherapy. It is hypothesized that LDHA, GPX3 and DOCK4 are new potential targets for lung adenocarcinoma, which can achieve breakthroughs in prognosis prediction, targeted prevention and treatment of lung adenocarcinoma and provide important guidance for anti-tumor.

## Introduction

As a leading cause of cancer-related morbidity and mortality, lung cancer accounts for approximately 20% of cancer-specific deaths worldwide^1^. Of these, non-small cell lung cancer (NSCLC) accounts for 85% of lung cancers, while lung adenocarcinoma (LUAD) accounts for half of all NSCLCs^2^. Currently, the overall survival (OS) of patients with LUAD remains poor, with a 5-year overall survival (OS) rate of 19%^3^ LUAD patients Exhibiting molecular and genetic heterogeneity^4^, ^5^. The In recent years, immunotherapy has shown significant efficacy in LUAD, but drug resistance and recurrence due to tumor heterogeneity still limit the efficacy of immunotherapy^6^, ^7^. The efficacy of immunotherapy is limited by the heterogeneity of tumors, resistance and recurrence. Therefore, we need to seek a new marker to develop new tools for predicting prognosis and immunotherapy efficacy to optimize personalized treatment strategies and improve patient survival.

G-protein-coupled receptors (GPCRs) are the largest family of cell surface signaling receptors known to play important roles in a variety of physiological functions, including tumor growth and metastasis^8^. GPCRs are the largest family of cell surface signaling receptors known to play important roles in multiple physiological functions including tumor growth and metastasis. In mammals, GPCRs comprise five major families, the largest being the Rhodopsin family, or class A, with approximately 284 members in humans (plus approximately 380 olfactory receptors), followed by the adhesion GPCRs family with 33 members, the glutamatergic family (class C), the secretin family (class B), and the convolvulus family. 22, 15, and 11 members, respectively^9^, ^10^. Studies have shown that GPCRs and their agonists are involved in growth stimulation of many solid tumors, including lung, colon, prostate, breast, and pancreatic cancers^11^. In addition, GPCRs dysregulation has been associated with a variety of human diseases and disorders including type II diabetes mellitus^12^. Alzheimer's disease^13^ , hypertension^14^ and heart Failure^15^. GPCRs also regulates proliferative signaling, replication immortality, growth inhibitor evasion, apoptosis resistance, angiogenesis initiation, and invasion and metastasis activation, which are thought to be hallmarks of cancer^16^. Recently, it has also been reported that several GPCRs members are associated with cancer progression and are frequently overexpressed in a variety of human cancers, including glioblastoma, colorectal cancer, and gallbladder cancer^17^-^21^. Multiple GPCRs are critical for tumor developmental processes, including tumor progression and survival^22^. Despite the growing evidence that GPCRs may play an important role in tumor biology, few studies have explored the potential of GPCRs in lung adenocarcinoma clinics, for example, as biomarkers for prognostic analysis of patients or for predicting patient response to immunotherapy, which represents an important area for future research.

In this study, we performed a comprehensive analysis of the expression of GPCR-related genes in LUAD based on bulk data and single-cell data from multiple datasets to establish and validate the signature of G protein-coupled receptor-related genes for the prediction of lung adenocarcinoma. A series of multi-omics systematic studies were also performed to better understand the molecular functions of GPCRs in this deadly malignancy. Our analysis suggests that GPCRRS is a prognostic model with good predictive efficacy.

### Research design

**Figure 1:**
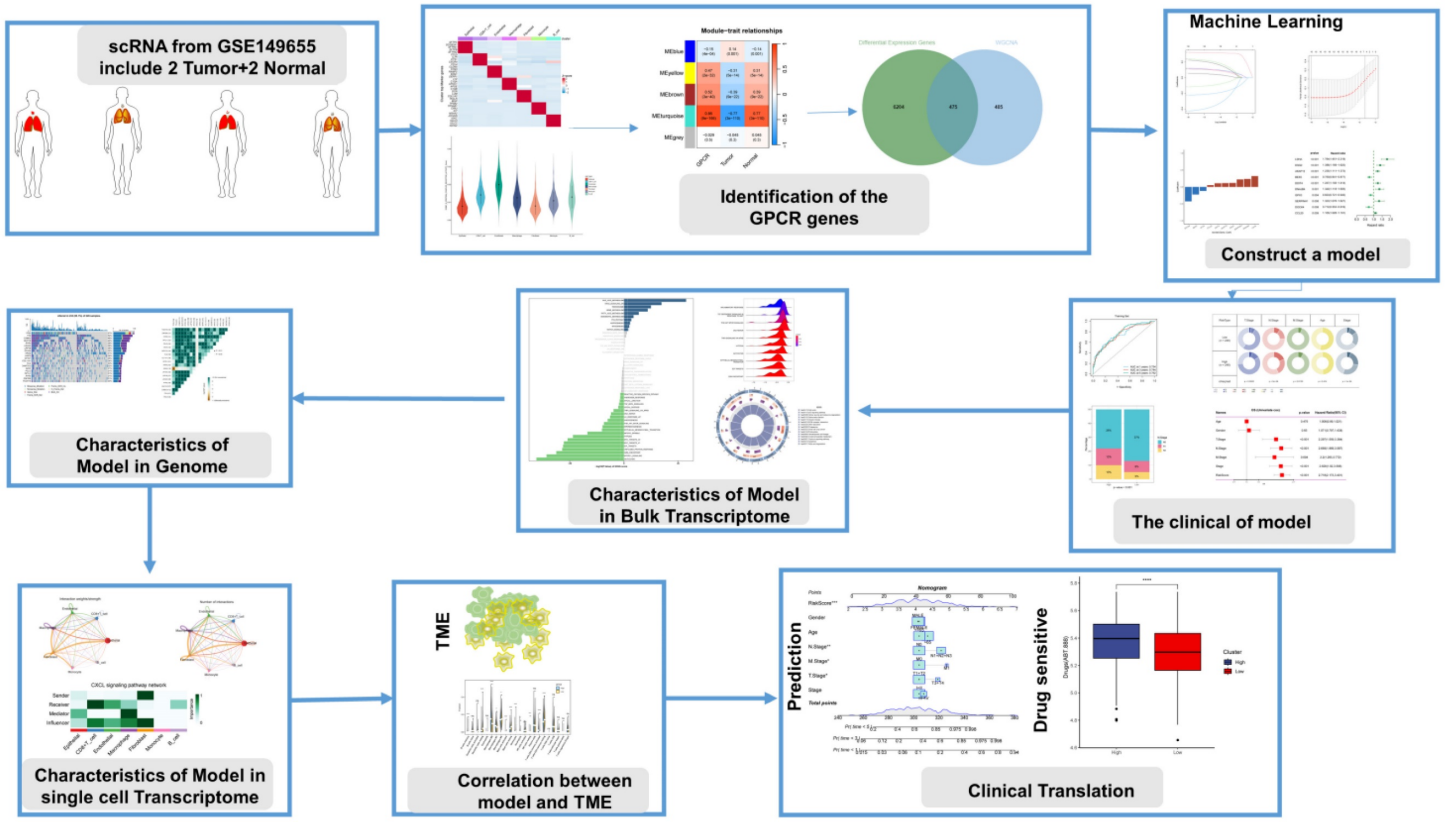
Flowchart of this study.

In this study, we investigated the characterization of G protein-coupled receptors (GPCRs) at a multi-omics level. We used single-cell and bulk data to identify GPCR-related genes, and then constructed a consistent G protein-coupled receptor-related signature (GPCRRS) using a novel machine learning framework that combines multiple machine learning algorithms and their combinations. To facilitate the application of GPCRRS, we evaluated whether GPCRRS could predict the occurrence, development and metastasis of LUAD. In addition, we constructed a GPCRRS nomogram to provide a quantitative tool for predicting the prognosis of each patient in clinical practice. The mechanisms of GPCRs were investigated at the bulk transcriptome, genome, and single-cell transcriptome levels, revealing that GPCRs are closely associated with the prognosis and immune status of LUAD. We further investigated the sensitivity of different risk subgroups to chemotherapeutic agents, including tyrosinase inhibitors, PARP inhibitors (ABT.888) and all-trans retinoic acid (ATRA). Our aim was to treat patients with lung adenocarcinoma. **Figure 1** provides a flowchart of our work.

## Materials and methods

### Data collection and processing

The analysis involved patients from two LUAD cohorts (GSE31210, GSE50081) and TCGA-LUAD. Patients without survival information and RNA sequencing (RNA-seq) data were excluded from the analysis. We used TPM data from TCGA for subsequent analysis. Construction of relevant prognostic features using 496 LUAD cases from the TCGA database, and the sample inclusion criteria for TCGA were 01A (Primary Tumor) type samples containing complete survival information. The training cohort was LUAD patients from TCGA, and the LUAD cohorts from the GEO dataset (GSE31210, GSE50081) represented the validation cohort for this study. In this case we used GSE31210 as the first validation set, which contains all tumour samples, and sample inclusion criteria for the GSE50081 integration cohort were samples with the histologic type of adenocarcinoma. To investigate the validity of prognostic features in predicting patient response to immunotherapy, we included the IMvigor210 cohort, which also included patients from the melanoma cohort from GSE78220 using the R package IMvigor210CoreBiologies^23^. Additionally to identify genes associated with GPCRs, we downloaded relevant data from the MSigDB database (http://www.gsea-msigdb.org/gsea/msigdb/index.jsp)^24^ (**Supplementary Table 1**).

### Single-cell RNA-seq analysis data collection and processing

We collected single-cell RNA sequencing data from LUAD patients from the GSE149655 dataset. We used the "Seurat" software package^25^. We analyzed the single-cell sequencing data. The data were first quality controlled (QC) by retaining cells with less than 10% mitochondrial genes and at least 3 cells expressing genes in the range of 200 - 8000. We identified highly variable genes for subsequent analysis with a variable gene count of 2000. We constructed clusters of cells using the "FindClusters" and "FindNeighbors" functions and visualized them using the "t-SNE" method. visualization. Finally, we annotate each cell cluster.

To elucidate the enrichment fraction of each cell in the scRNA-seq dataset, we used the "AUCell" package^26^. We used the "FindMarkers" function in the Seurat package to analyze the differentially expressed genes (DEGs) between the two groups, in which the statistical significance of the differentially expressed genes (DEGs) was calculated using the Wilcoxon test (p. adj < 0.05). Genes differentially expressed between cells with high and low GPCRs scores at the single-cell transcriptome level were considered to be involved in GPCRs. these genes were subsequently included in the bulk transcriptome data of WGCNA for analysis. We also used the R package "CellChat"^27^ to perform cell-to-cell communication analysis.

### Gene set variation analysis, Single-sample gene set enrichment analysis (ssGSEA) and gene set enrichment analysis (GSEA)

ssGSEA is a widely used method to quantify the enrichment score for a specific set of genes in a single sample. The ssGSEA score for each sample reflects the extent to which a particular gene set is systematically up- or down-regulated in the sample. In this study, we utilized the R package "GSVA"^28^ in ssGSEA to obtain GPCRs scores for each TCGA-LUAD sample. In addition, to identify potential pathways associated with the feature, we used the "limma" package^29^. We analyzed the pathways with significant differences between high- and low-risk groups and calculated GSVA scores for 50 signature pathways. To reveal the biological processes (BP), cellular components (CC) and molecular functions (MF) involved in different risk subgroups, we used the R package "clusterProfiler"^30^. which analyzed the KEGG and HALLMARK gene sets between the two risk subgroups using the criteria of FDR < 0.25 and |NES|> 1, for Gene Set Enrichment Analysis (GSEA).

### Identification of G protein-coupled receptor-related signature genes using WGCNA

WGCNA analysis is a systematic biological approach that can characterize patterns of genetic associations between different samples and has the ability to identify highly covariant sets of genes. In our study, we used the R package "WGCNA"^31^ that performs WGCNA analysis based on TCGA-LUAD bulk RNA-seq data. Initially, a suitable soft threshold β is calculated to fulfill the criteria for constructing scale-free networks. Then, we converted the weighted neighbor-joining matrix to topological overlap matrix (TOM) and calculated the dissimilarity (dissTOM). For gene clustering and module identification, we applied the dynamic tree-cutting method. Finally, the modules with the highest correlation with GPCRs were identified for subsequent analysis.

### Machine learning to build prognostic models

In the TCGA-LUAD bulk RNA-seq data, we used the R package DESeq2^32^ for differential analysis of normal and tumor samples (|logFC|> 1 and p.adj < 0.05). We then crossed DEGs at the bulk RNA-seq level with genes in the GPCRs-associated modules identified by WGCNA. These crossover genes were considered to be involved in G protein-coupled receptors at both the bulk and single-cell transcriptome levels, and therefore, we termed them G protein-coupled receptor-related genes (GPCR-related genes).

### G protein-coupled receptor-related signatures

First, we applied one-way Cox regression analysis to screen GPCRs-related genes with potential prognostic role in the TCGA cohort. The TCGA cohort was considered as the training set, while the GSE31210 and GSE50081 cohorts were set as the external validation set. Lasso, Ridge,stepwise Cox, CoxBoost, random survival forest (RSF), elastic net (Enet), partial least squares regression for Cox (plsRcox), supervised principal components (SuperPC), generalized boosted regression modeling (GBM) and survival support vector machine (survival-SVM). and other 10 machine learning algorithms. We arranged multiple machine learning combinations of these 10 algorithms in the TCGA queue for variable selection and model construction based on the tenfold cross-validation framework. All constructed models are validated on the TCGA training set and the GSE31210 and GSE50081 datasets. For each model, we calculate its C-index from the training and validation sets. We then ranked the predictive performance of the models based on the average C-index. We selected the combination of algorithms with robust performance and clinical translational significance. As a result, we built a final signature that predicts the overall survival of LUAD patients, called the G protein-coupled receptor-related signature (GPCRRS).

### Survival analysis and construction of a predictive nomogram

In this study, TCGA-LUAD, GSE31210 and GSE50081 groups were categorized into high and low risk groups using the median risk score. Subsequently, we performed KM survival curve analysis using the R software package "survminer" to investigate whether there were significant differences in overall survival (OS), progression-free survival (PFS), and disease-specific survival (DSS) between the high- and low-risk groups (log-rank test, p < 0.05). In addition, we performed ROC curve analysis using the "timeROC" software package to assess the sensitivity and specificity of GPCRRS in predicting OS in LUAD patients^33^. We also compared the sensitivity and specificity of GPCRRS in predicting OS in LUAD patients. We also compared the area under the curve (AUC) of GPCRRS with other clinical characteristics. In addition, we explored the correlation between GPCRRS and clinical characteristics such as age, gender, staging, T, M, and N. We performed univariate and multivariate Cox regression analyses on the TCGA-LUAD, GSE31210, and GSE50081 datasets to determine whether GPCRRS was an independent prognostic factor predicting survival in patients with LUAD. To improve the prognostic accuracy and predictive power of our model, we developed a nomogram combining GPCRRS and clinical characteristics of expected survival of LUAD patients. Finally, we assessed the precision discrimination and accuracy of the nomogram using ROC curves, C-indexes, and calibration curves, and assessed the net clinical benefit of the nomogram using decision curve analysis (DCA).

### Mutation and drug susceptibility analysis

Generation of Mutation Annotation Format (MAF) in the TCGA database using the R package "maftools" to map somatic mutations in LUAD in the low-risk and high-risk groups^34^. Tumor mutation burden (TMB) was also calculated for each LUAD patient in the TCGA cohort. Drug sensitivity analysis was performed using the R package "pRROphetic"^35^. The R package "pRROphetic" was used for drug sensitivity analysis. A council plot was developed using the HIPLOT website (https://hiplot.com.cn/) to demonstrate drug sensitivity in low- and high-risk populations.

### TME landscape analyses

Enrichment scores for infiltrating immune cells and immune function were calculated and compared using single sample gene set enrichment analysis (ssGSEA)^23^, ^36^. The ESTIMATE algorithm was used to calculate immune scores, ESTIMATE scores and stromal scores between the two groups^37^. TIDE score data were obtained from the TIDE website (http://tide.dfci.harvard.edu/).

### Immunotherapy datasets and TCIA

Anti-PD-1 or anti-PD-L1 checkpoint inhibition therapies are receiving increasing attention as an important component of immunotherapy. To assess the performance of risk profiles in predicting response to immunotherapy (immune checkpoint blockade), we collected transcriptomic data from the IMvigor210 cohort of patients treated with anti-PD-L1 therapy as well as corresponding clinical data. We also downloaded transcriptomic data from the GSE78220 cohort, which included melanoma patients who received anti-PD-1 checkpoint inhibition therapy prior to treatment.

To determine immunogenicity based on immunomodulators, immunosuppressive cells, MHC molecules, and effector cells, we used the Immunophenoscore (IPS) algorithm, which calculates IPS scores based on unbiased gene expression of representative cell types using a machine learning approach. Higher IPS scores indicate a better response to immunotherapy. IPS scores of TCGA-LUAD patient samples were obtained from The Cancer Immunome Atlas (TCIA) database (https://tcia. at//home). Immunotherapy response was then predicted using the SubMap online tool^38^.

### Cell line culture and qRT-PCR

All cells were cultured at 37°C in an incubator with 5% CO2 atmosphere. Normal human liver cell line 2B, lung adenocarcinoma cells H1299 and A549 were obtained from the Chinese Academy of Sciences (Shanghai, China). Cell culture media, plates and dishes were from Thermo Fisher Scientific (Invitrogen, USA) and Corning Inc. 2B cells, H1299 cells and A549 cells were detached and inoculated into 60 mm dishes overnight at an initial density of 1 × 106 cells/well. Subsequently, SYBR Green qPCR mix (Vazyme, China) was used to synthesize cDNA for real-time PCR. Our results were analyzed using the comparative Ct method and the Ct values of each gene were normalized by the Ct reads of the corresponding GAPDH. All data are expressed as mean ± standard deviation (SD) of three independent experiments, and primer sequences are shown (**Supplementary Table 2**).

### Statistical analysis

The statistical analysis of this study was performed using R4.0.1 software. For quantitative data, the statistical significance of normally distributed variables was estimated by the Student's *t* test, and non-normally distributed variables were analyzed using the Wilcoxon software. Comparisons between more than two groups were made using the Kruskal-Wallis test and oneway analysis of variance as non-parametric and parametric methods, respectively. Kaplan-Meier survival analysis was performed with the R package "Survminer". Statistical significance was set as P< 0.05. RT-qPCR results were analyzed using a Student's *t* test. Unless otherwise stated, statistical significance was set at p<0.05.

## Results

### G protein-coupled receptor characterization of the single-cell transcriptome

**Figure 2:**
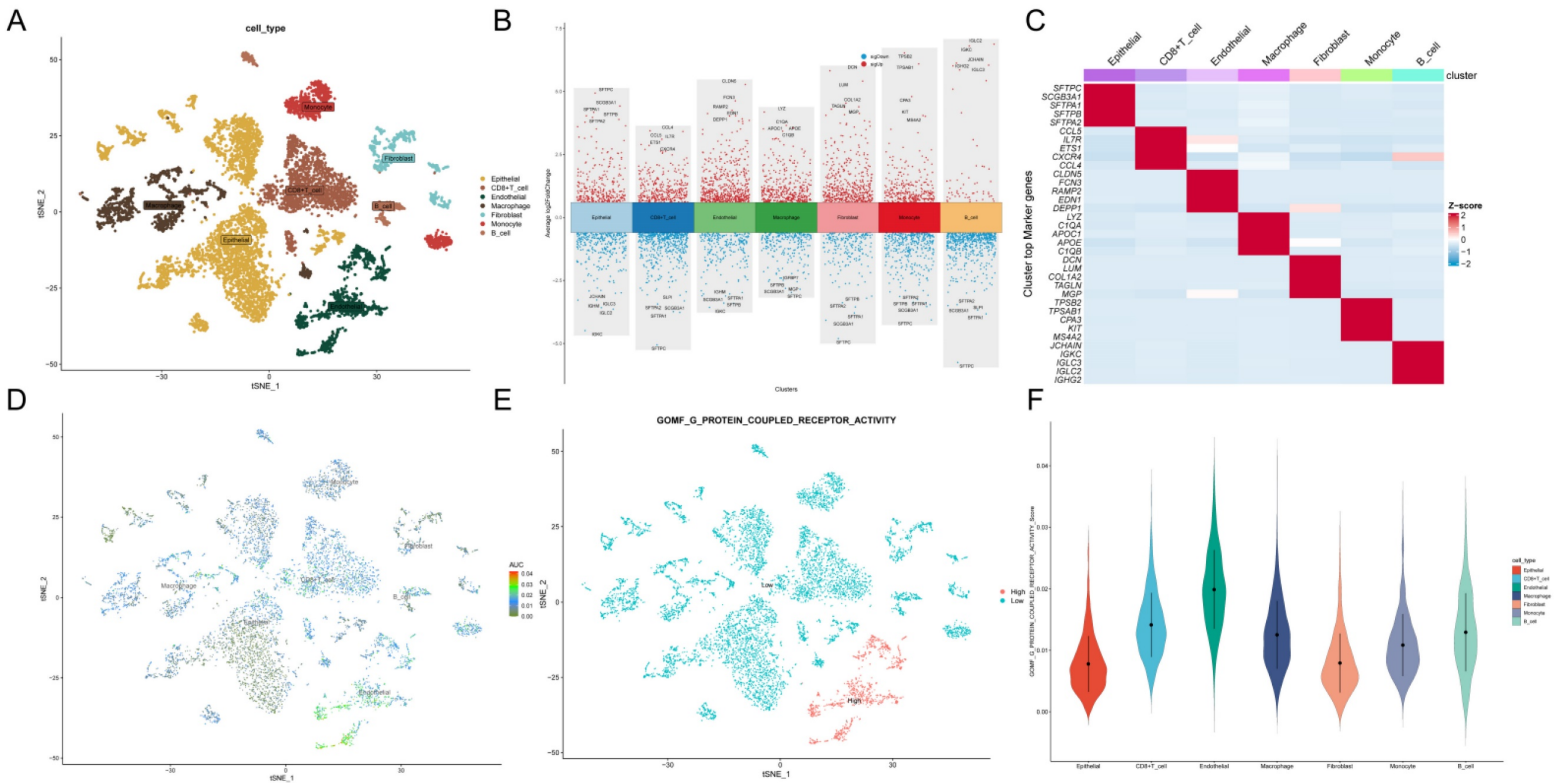
** G protein-coupled receptor characterization of the single-cell transcriptome.** A: t-SNE plot showing cell types identified by marker genes. B: volcano plot showing the 5 most important marker genes in each cell cluster. C: heat map showing the 5 most important genes in each cell. D: G protein-coupled receptor (GPCR) scores for each cell. E: Distribution of high and low groupings of GPCR scores across cell types. F: violin plot showing GPCR scores in each cell

We obtained data from 2 normal and 2 LUAD patients from the GSE149655 single-cell dataset, and a total of 8954 cells were obtained from the scRNA-seq data after initial screening. The first 2000 variant genes were then subjected to principal component analysis (PCA) and t-distribution random neighbor embedding (t-SNE) downscaling. Clustering was then performed to aggregate all cells into 15 clusters with a resolution of 0.8. Cells were then annotated and labeled into seven major clusters, namely endothelial cells, CD8+ T cells, epithelial cells, macrophages, monocytes, B cells and fibroblasts (**Figure 2A**). Volcano and heat maps showed the top five labeled genes for each cell cluster (**Figure 2B, C**).

To assess G protein-coupled receptors (GPCRs) activity in different cell types, we used the AUcell R package to calculate the expression levels associated with GPCRs in all cells (**Figure 2D**). Among these seven cell types, we observed significantly elevated GPCRs activity in endothelial cells (**Figure 2E, F**). Based on the GPCRs activity, we classified the cells into high and low GPCRs groups and identified differentially - expressed genes (DEGs) between the two groups for further analysis (**Supplementary Table 3**).

### Identification and key genes of G protein-coupled receptors in bulk RNA-seq

**Figure 3:**
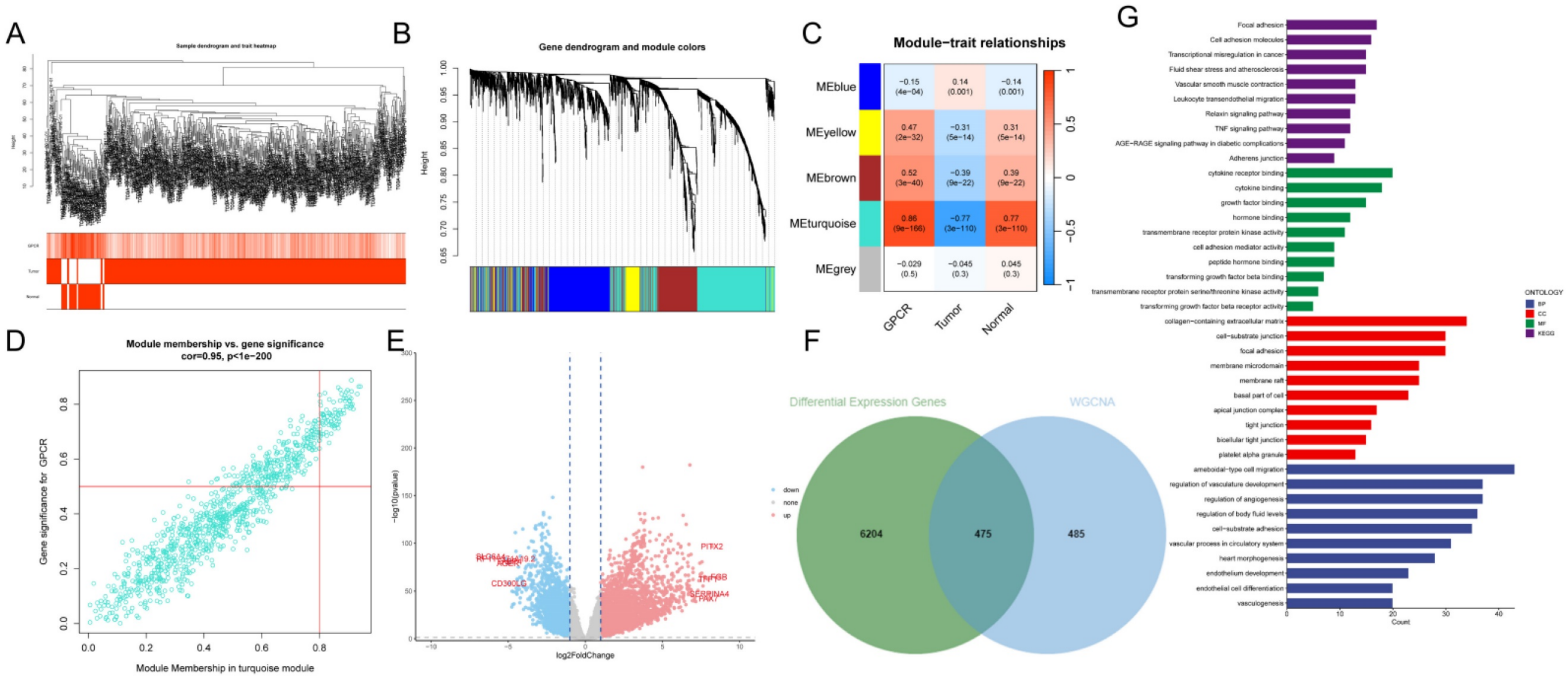
** Identification of G protein-coupled receptor-related genes (GPCRRgenes).** A: Dendrogram showing hierarchical clustering of TCGA-LUAD samples. The heatmap at the bottom indicates the GPCR score of each sample, calculated by the ssGSEA algorithm. B: Clustering dendrogram for WGCNA analysis. C: Module-trait heatmap showing that the MEturquoise module is strongly associated with the GPCR trait. D: Gene significance (GS) in the turquoise module versus module affiliation (module membership, MM) relationship scatterplot. E: Volcano plot showing the results of differential analysis of TCGA-LUAD tumor samples versus normal samples, specifically marking the top 5 genes that were up or down regulated. F: Venn plot showing the crossover between MEturquoise modules and DEGs in bulk-RNA-seq genes. G: GO and KEGG enrichment of GPCRRgenes.

In this study, we used ssGSEA to score G protein-coupled receptors (GPCRs) for each TCGA cohort sample as clinical phenotypic data for WGCNA analysis. To identify genes associated with G protein-coupled receptors, we performed WGCNA on differential genes derived from from the single-cell dataset to identify genes associated with G protein-coupled receptors (**Figure 3A**). To ensure that the topological network was scale-free, the optimal soft threshold for power was chosen = 4 (**Supplementary Figure. 1A**). A total of 5 modules were obtained by setting the minimum module gene count to 60 and medissres to 0.25 (**Figure 3B**).

The results showed that the MEturquoise module was strongly correlated with the GPCRs score in bulk-RNA-seq (cor = 0.86, **Figure 3C**). In addition, the scatter plot of gene significance (GS) versus module membership (MM) of the turquoise module showed a significant correlation (cor = 0.95, p = 1e-200, **Figure 3D**), suggesting that the genes in the turquoise module may have functional significance in relation to G protein-coupled receptors.

The volcano plot (**Figure 3E**) shows the differentially expressed genes (DEGs) between tumor and normal lung tissues in TCGA-LUAD bulk RNA-seq (|logFC|> 1 and p.d adj < 0.05). We crossed the genes in the blue module with DEGs from TCGA-LUAD-bulk RNA-seq and finally identified 475 genes (**Figure 3F**). These genes were considered G protein-coupled receptor-related genes (GPCRR genes), which were involved in GPCRs at the bulk-RNA-seq and single-RNA-seq levels. GO and KEGG enrichment for GPCRR genes analysis (**Figure 3G**) showed that GPCRs genes in KEGG analysis, including adhesion, cell adhesion molecules, transcriptional dysregulation in cancer, transendothelial migration of leukocytes, TNF signaling pathway, and molecular functions (MFs), such as cytokine receptor binding, cytokine binding, and transmembrane receptor protein kinase activity, as well as cellular components (CCs), including collagen-containing extracellular matrix were significantly enriched, as well as biological processes (BP) such as cell migration, regulation of vascular system development and cell-substrate adhesion.

### Machine learning to build prognostic models

**Figure 4:**
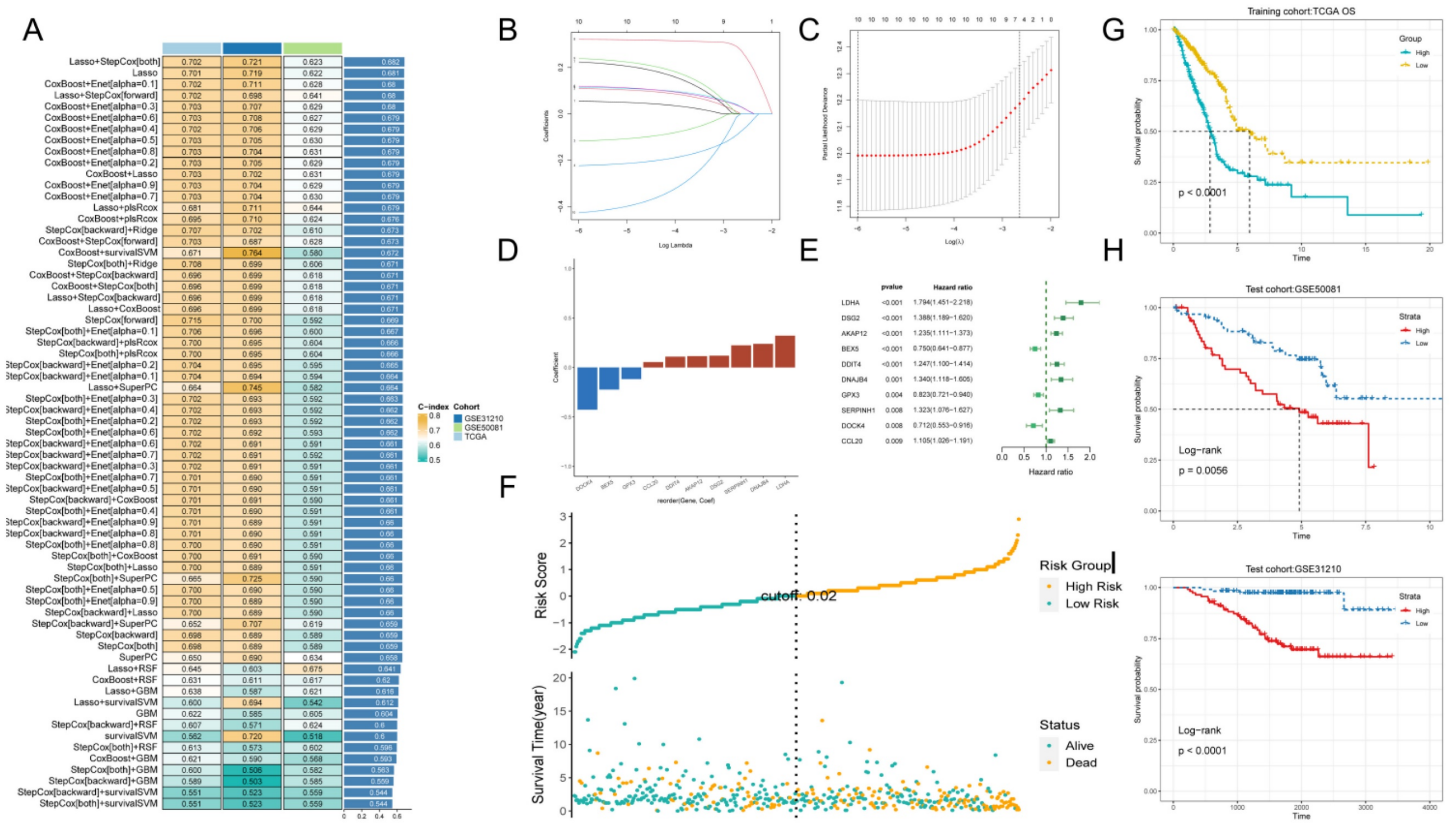
** Machine learning to build prognostic models.** A: Multiple machine learning is utilized through a ten-fold cross-validation framework and the C-index is further calculated for each model on all validation datasets. B, C: Visualization of LASSO regression in the TCGA-LUAD cohort. D: Stepwise Cox regression to obtain the regression coefficients for 10 genes. E: Forest plot showing the results of stepwise Cox regression. F: Distribution of risk scores and overall survival status of patients in the TCGA-LUAD cohort.G-I: Based on the log-rank test, the Kaplan-Meier curve of OS was obtained based on the GPCRRS in the TCGA training and external validation sets, GSE31210 and GSE50081.

Next, we performed one-way regression analysis (p < 0.01) on 475 GPCRR genes and found 62 genes. To further validate and construct the model, we cross-checked the TCGA-LUAD dataset with GSE31210 and GSE50081 and obtained 59 common genes. To construct a consistent G protein-coupled receptor-related signature (GPCRRS), we utilized multiple machine learning algorithms to analyze the 59 prognostic genes obtained from univariate Cox regression analysis. The TCGA-LUAD dataset was used as the training set. In the training set, we fitted multiple prediction models with a ten-fold cross-validation framework and calculated the C-index for all training and validation sets (**Figure 4A**).

Among multiple machine learning models, we choose the prediction model with the first ranked average C-index, while it shows good prediction ability in the training set validation and external validation set. We therefore choose Lasso + StepCox [both] (**Figure 4A**). As a result of the comprehensive screening, we found Lasso + StepCox[both] to be a predictive model with high accuracy. Using a tenfold cross-validation framework, by minimizing the partial likelihood bias (**Figure 4B, C**), we identified the optimal λ value in the LASSO analysis (**Figure 4B, C**). Genes with nonzero coefficients in the LASSO analysis were then analyzed by stepwise Cox proportional risk regression [both], and 10 genes were finally identified (**Figure 4E**). Subsequently, we calculated the risk score for each patient by weighting the expression of the 10 genes with the regression coefficients in the Cox model (**Figure 4D**), which allowed us to categorize all the patients into high-risk and low-risk groups based on the median risk score. Notably, the number of patients experiencing death increased progressively as the risk score increased (**Figure 4F**). Moreover, the datasets in the training set and external validation set showed that the overall survival (OS) of patients in the high-risk group was significantly lower than that of the low-risk group (p < 0.001, log-rank test; **Figure 4G-I**).

### Development and validation of a risk model

**Figure 5:**
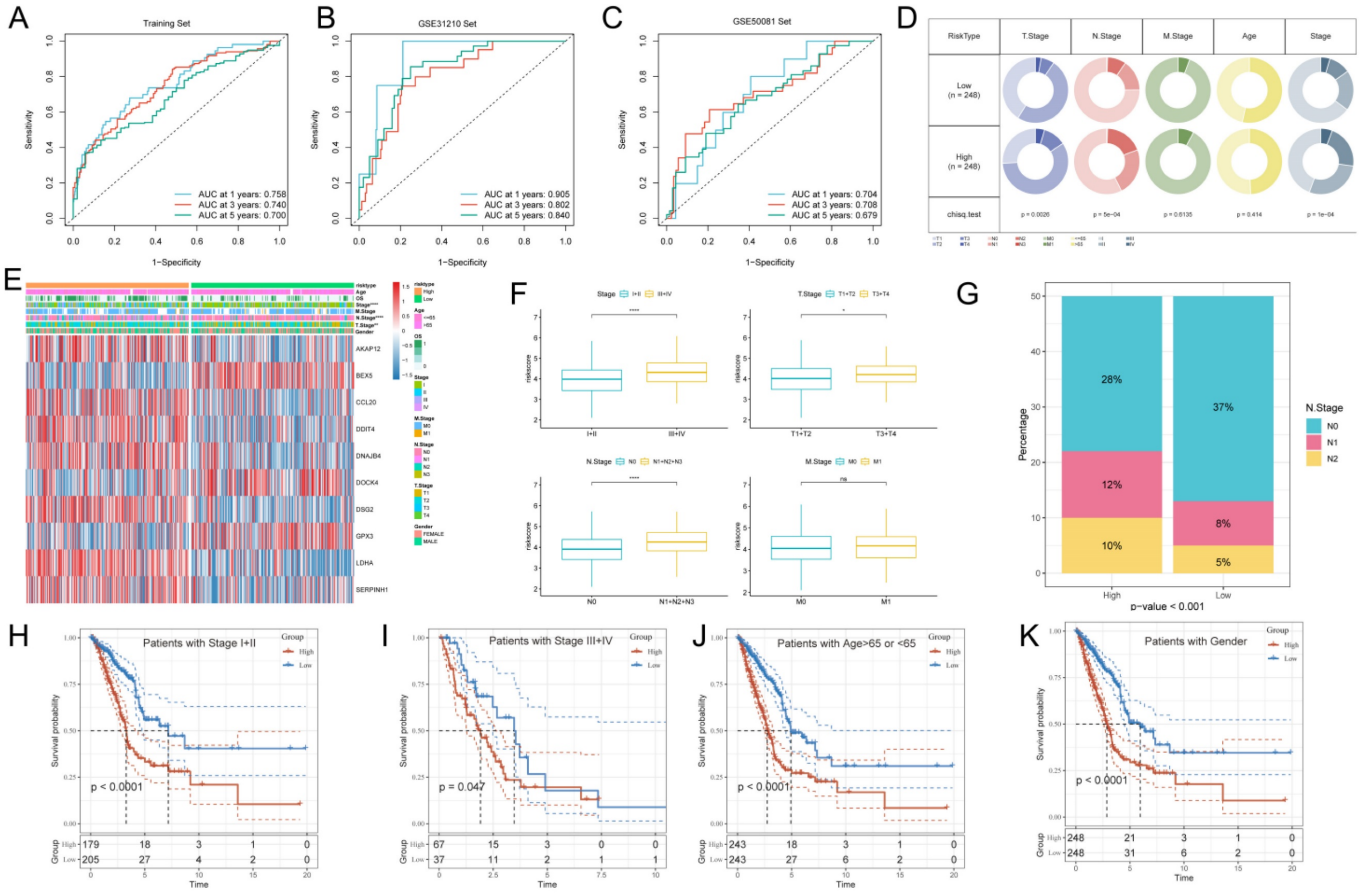
** Development and validation of a risk model. Evaluation of the GPCRRS model.** A-C: ROC curves showing the specificity and sensitivity of GPCRRS in predicting 1-, 3-, and 5-years OS in the TCGA training set (A), GSE31210 external validation set (B), and GSE50081. D: Correlation of GPCRRS low-risk and high-risk groups with clinical features. E: Distribution of clinical features and modeled gene expression according to GPCRRS risk scores. F: Differences in risk scores of staged, T, N, and M patients. G: Proportion of N stage in GPCRRS risk subgroups. H-K: Kaplan-Meier curves showing that GPCRRS was stable in the subgroup of LUAD patients. Stable performance in the subgroup of LUAD patients, including age and stage.

The ROC curve analysis showed that the area under the curve (AUC) of GPCRRS was 0.758, 0.740, and 0.700 for the 1-, 3-, and 5-years training sets, respectively; 0.905, 0.802, and 0.840 for the external GSE31210 validation set; and 0.704, 0.708, and 0.679 for the GSE50081 dataset (**Figure 5A-C**). These results indicate that GPCRRS has a strong discriminatory ability. In addition, we compared the AUC of GPCRRS with other clinical features (including age, gender, T, N, M, and total stage), and the results showed that the AUC of GPCRRS was significantly better than other clinical features (**Supplementary Figure 1B**). In addition, we assessed the correlation between GPCRRS and various clinical features. In the TCGA-LUAD dataset, we observed significant differences in staging, T and N staging between the high- and low-risk groups (P<0.05, chi-square test) (**Figure 5E, F**). In addition, we noticed that the risk scores of patients with stage III+IV, T3+4 and N1+2+3 were significantly higher than those of patients with stage I-II, T1+2 and N0 (P<0.05, Wilcox test). Lymph node metastasis is prone to malignant progression of lung adenocarcinoma, and lymph node metastasis is closely related to N stage. We found a significant difference in the proportion of N stage in different risk groups (**Figure 5G**). By KM curve analysis, we also found that GPCRRS showed good prognostic ability in subgroups with different clinical characteristics (including age, gender, grading, T, N, and M) (**Figure 5H-K**, **Supplementary Figure 1C-F**). As shown in Table 1, GPCRRS was also significantly associated with T, N staging and tumor stage. These results suggest that GPCRRS is associated with poor prognosis in LUAD patients.

### Survival analysis and construction of a predictive nomogram

**Figure 6:**
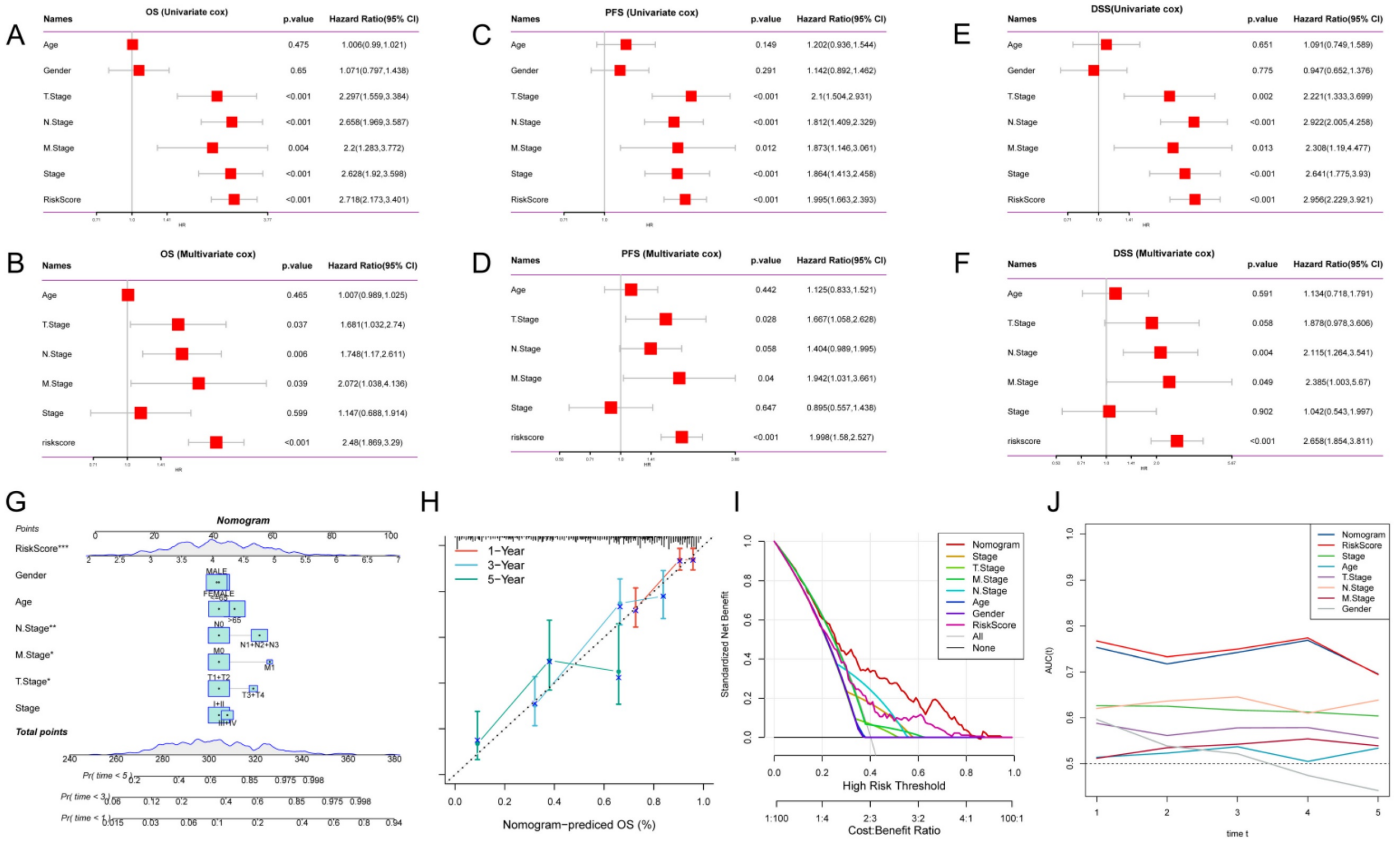
** Survival analysis and construction of a predictive nomogram.** A-F: Univariate and multivariate analyses of clinical characteristics and GPCRRS for OS (A, B), PFS (C, D), and DSS (E, F) in the TCGA-LUAD cohort. G: Construction of a nomogram according to the GPCRRS and clinical characteristics to construct nomograms including age, gender, stage, T, N, M. H: Column line graph calibration curves for 1, 3 and 5 years OS. I: Decision curve analysis (DCA) to show the net benefit by applying the nomogram and other clinical characteristics. J: Assessment of the predictive power of the column line graphs and clinicopathological characteristics by TIME-ROC analysis.

To assess whether GPCRRS was an independent prognostic factor for LUAD, we performed univariate and multivariate Cox regression analyses for OS, PFS, and DSS in the TCGA-LUAD dataset (**Figure 6A-C**). Our findings showed that GPCRRS was a significant risk factor for OS, PFS and DSS in univariate analysis (HR > 1, p < 0.001). Furthermore, in multifactorial analysis showed that GPCRRS remained a significant risk factor for OS (HR=2.48, CI:1.869-3.29 p < 0.001), PFS (HR=1.998, CI:1.58-2.527, p < 0.001) and DSS (HR=2.658, CI: 1.854-3.811, p < 0.001) independent prognostic factors, suggesting a strong prognostic ability in LUAD patients (**Figure 6D-F**).

To validate the predictive role of GPCRRS in the clinical setting, we constructed a nomogram based on GPCRRS and clinical features (**Figure 6G**). The calibration curves showed good agreement between the nomogram predictions and the actual observations (**Figure 6H**). Decision curve analysis (DCA) showed better predictive efficacy of the nomogram compared with other clinical features (**Figure 6I**). TimeROC analysis in the TCGA cohort confirmed that the AUCs of the column line graph and risk scores exceeded those of other metrics (**Figure 6J**). These results suggest that the nomogram diagnostic method based on GPCRRS provides a reliable and accurate tool for personalized prognostic prediction in LUAD patients.

### Pathway enrichment analysis of G protein-coupled receptors in bulk transcriptome

**Figure 7:**
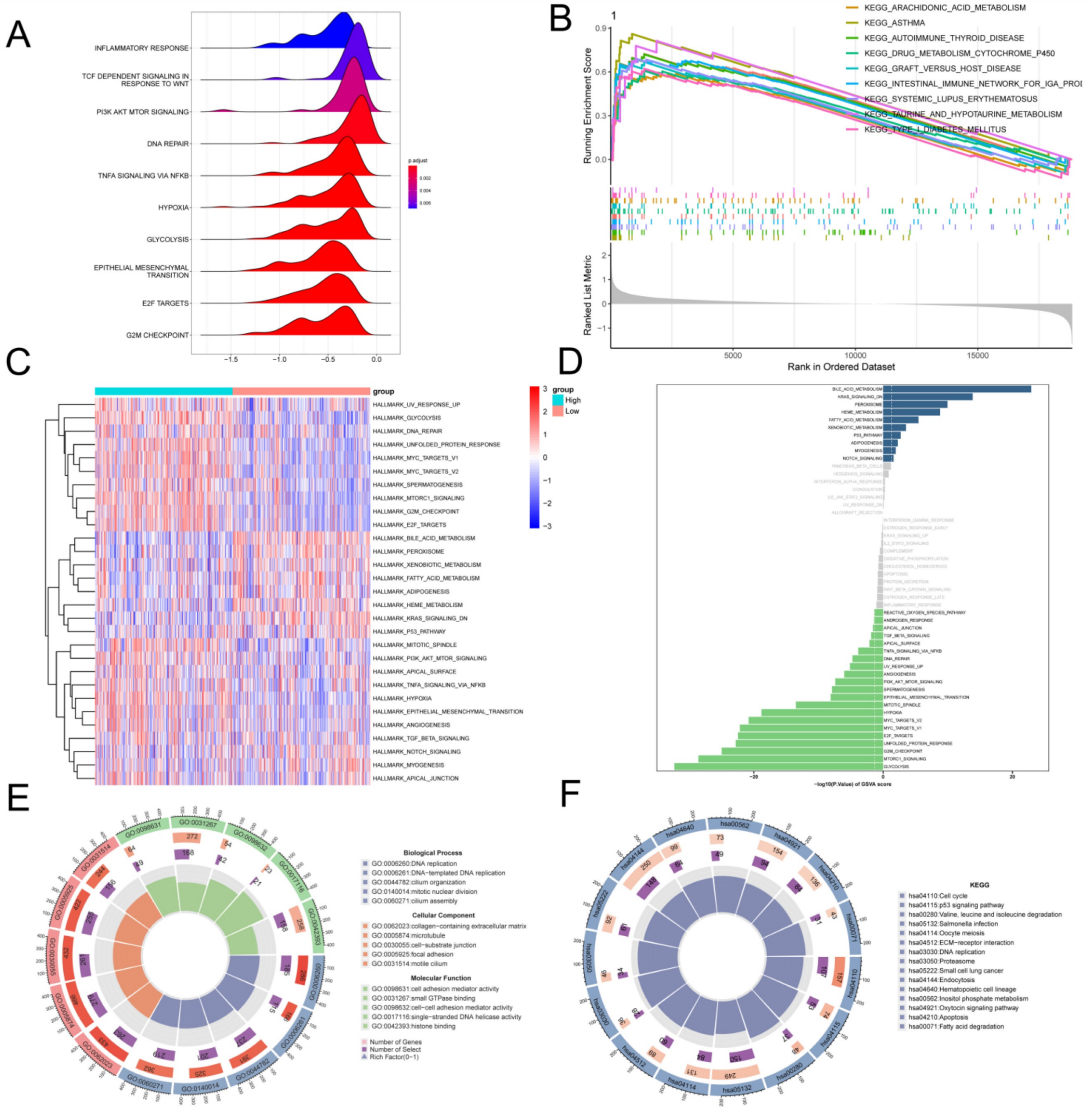
** Pathway enrichment analysis of G protein-coupled receptors in bulk transcriptome.** A: Ridge diagram showing pathway analysis of high-risk group; B: GSEA analysis showing KEGG term enrichment in low-risk group; C: Difference in HALLMARK pathway activity between high- and low-risk of GSVA score; D: Correlation between risk score and HALLMARK pathway activity of GSVA score. E: Correlation between G pathway activity of high- and low-risk differential genes; F: KEGG enrichment analysis of high and low risk differential genes.

To further investigate the molecular mechanisms underlying the association between GPCRRS and LUAD prognosis, we performed gene set enrichment analysis. In the GSEA analysis based on the REACOME gene set, we observed that the high-risk group was enriched in the positive regulation of TCF_DEPENDENT_SIGNALING_IN_RESPONSE_TO_WNT, TNFA SIGNALING_VIA_NFKB, and E2F_TARGETS (**Figure 7A**), while the low-risk group was enriched in the positive regulation of the REACOME gene set based on the c2.cp.kegg.v2023.1.Hs.symbols gene set was mainly enriched with the positive regulation of KEGG-AUTOIMMUNE-THYROID-DISEASE, KEGG-ASTHMA and KEGG-ARACHIDONIC-ACID-METABOLISM (**Figure 7B**). GSVA enrichment Analysis based on the HALLMARK gene set demonstrating differential pathways between the two revealed that the high-risk group was mainly highly expressed in E2F-TARGETS, G2M-CHECKPOINT, TNFA_SIGNALING_VIA_NFKB, and MYC_ TARGETS_V2, which may imply that the high-risk group was oncogenic through these pathways; the low-risk group was mainly expressed in the BILE_ACID_METABO-LISM, FATTY_ACID_METABOLISM showed higher activity (**Figure 7C, D**). GO and KEGG enrichment analyses demonstrated the enrichment analysis of the two differential genes, GO was mainly enriched for DNA replication, collagen- containing extracellular matrix, cell adhesion mediator activity; KEGG enrichment analysis demonstrated ECM-receptor interaction, Fatty acid degradation, Apoptosis, etc. (**Figure 7E, F**). This suggests that GPCRRS is closely associated with cancer-related biological processes and metabolic pathways.

### Tumor mutation analysis in bulk RNA-seq

**Figure 8:**
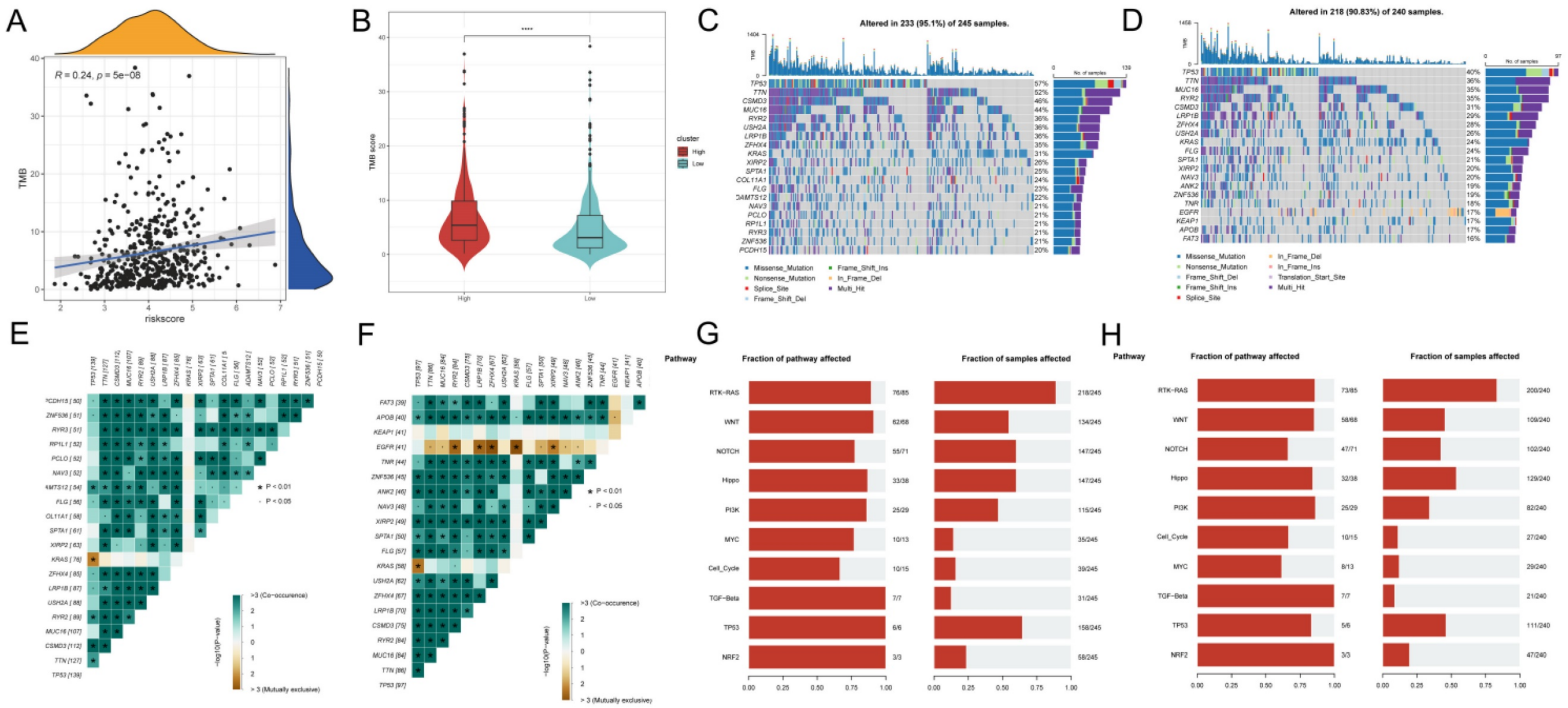
** Tumor mutation analysis.** A: Risk score and TMB correlation analysis. B: Difference in TMB between low-risk and high-risk groups. C-D: Mutation waterfall map between two groups, (C) high-risk group; (D) low-risk group. E-F: Correlation of the first 20 mutated genes between two groups, (E) high-risk group, (F) low-risk group; G-H: Pathways regulated by mutated genes in different risk groups, (G) high-risk group, (H) low-risk group.

To investigate the correlation between risk scores and TMB, Spearman correlation analysis was performed in this study, and a significant positive correlation was found between risk scores and TMB (R = 0.24, P< 0.001, **Figure 8A**). Patients in the high-risk group had higher TMB levels than those in the low-risk group (**Figure 8B**). In addition to investigate the differences in genomic mutations between GPCRRS subgroups, we depicted a waterfall plot between the high-risk and low-risk groups, and we found distinct mutation profiles between the two risk groups. (**Figure 8C, D**). In addition, we analyzed the correlation of the top 20 mutations between the two groups (**Figure 8E, F**). Subsequently, we investigated the mutation frequencies of the 10 major oncogenic pathways in the two risk subtypes. Our results showed that most of the oncogenic mutation pathways were detected in the high-risk types, including RTK-RAS, WNT, NOTCH, MYC and TP53 pathways (**Figure 8G, H**).

### Characterization of G protein-coupled receptors in single cells

**Figure 9:**
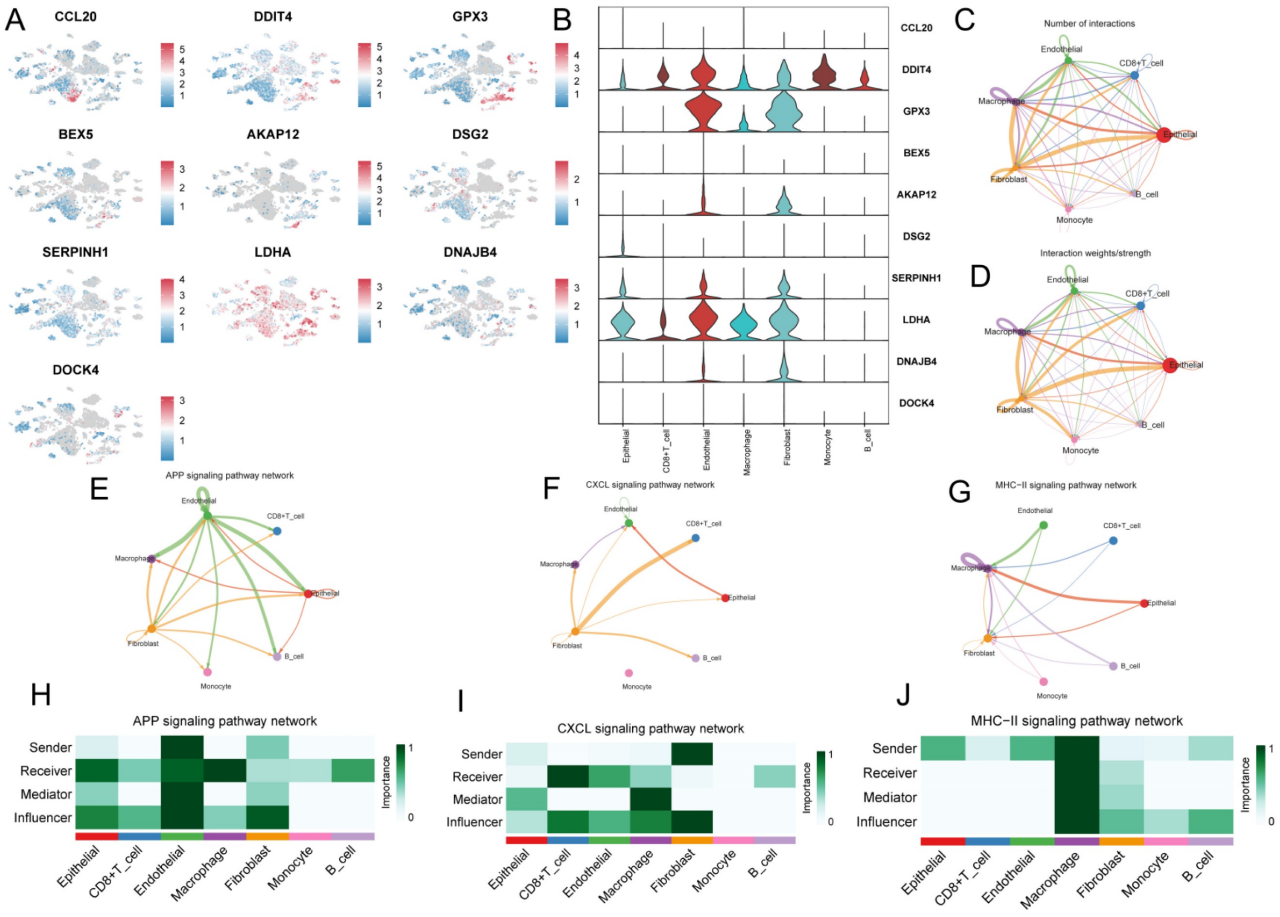
** Characterization of G protein-coupled receptors in single cells.** A: CCL20, DDIT4, GPX3, BEX5, AKAP12, DSG2, SERPINH1, LDHA were analyzed by single cell RNA-seq, DNAJB4 and DOCK4 expression in different cell types. B: Violin plots of CCL20, DDIT4, GPX3, BEX5, AKAP12, DSG2, SERPINH1, LDHA, DNAJB4, and DOCK4 genes demonstrating. C: Quantitative intercellular communication; D: Intense intercellular communication. E-F: Circos plots showing APP (H), CXCL (I), and MHC-II (J) signaling pathway networks; H-J: heatmaps showing the roles played by different cell types in the pathway networks.

To investigate the role of GPCRRS in the tumor microenvironment (TME) at the single-cell transcriptome level, we analyzed the expression patterns of CCL20, DDIT4, GPX3, BEX5, AKAP12, DSG2, SERPINH1, LDHA, DNAJB4, and DOCK4 in different cell types (**Figure 9A**). The results showed that these genes were mainly expressed in immune cells such as macrophages, endothelial cells and fibroblasts (**Figure 9B**).

Next, we investigated the role of intercellular communication, and we found that fibroblasts dominated the communication component (**Figure 9C, D**). In addition, we studied their interactions with other cell types in the TME. We found that different immune cells have different communication patterns (**Figure 9E-J**). different types of cells in the TME can play the roles of senders, receivers, mediators, and influencers in cellular communication, leading to the generation of specific signals between cells. The results showed that fibroblasts, macrophages, and endothelial cells communicate with more types of TME cells and play stronger roles as mediators and influencers in the APP signaling pathway, CXCL signaling pathway, and MHC-II signaling pathway. These signaling pathways can regulate adhesion, differentiation, and metastasis and influence cancer cell survival^39^, ^40^ (**Figure 9H-M**).

### Immunoscape and immunologic properties of G protein-coupled receptors

**Figure 10:**
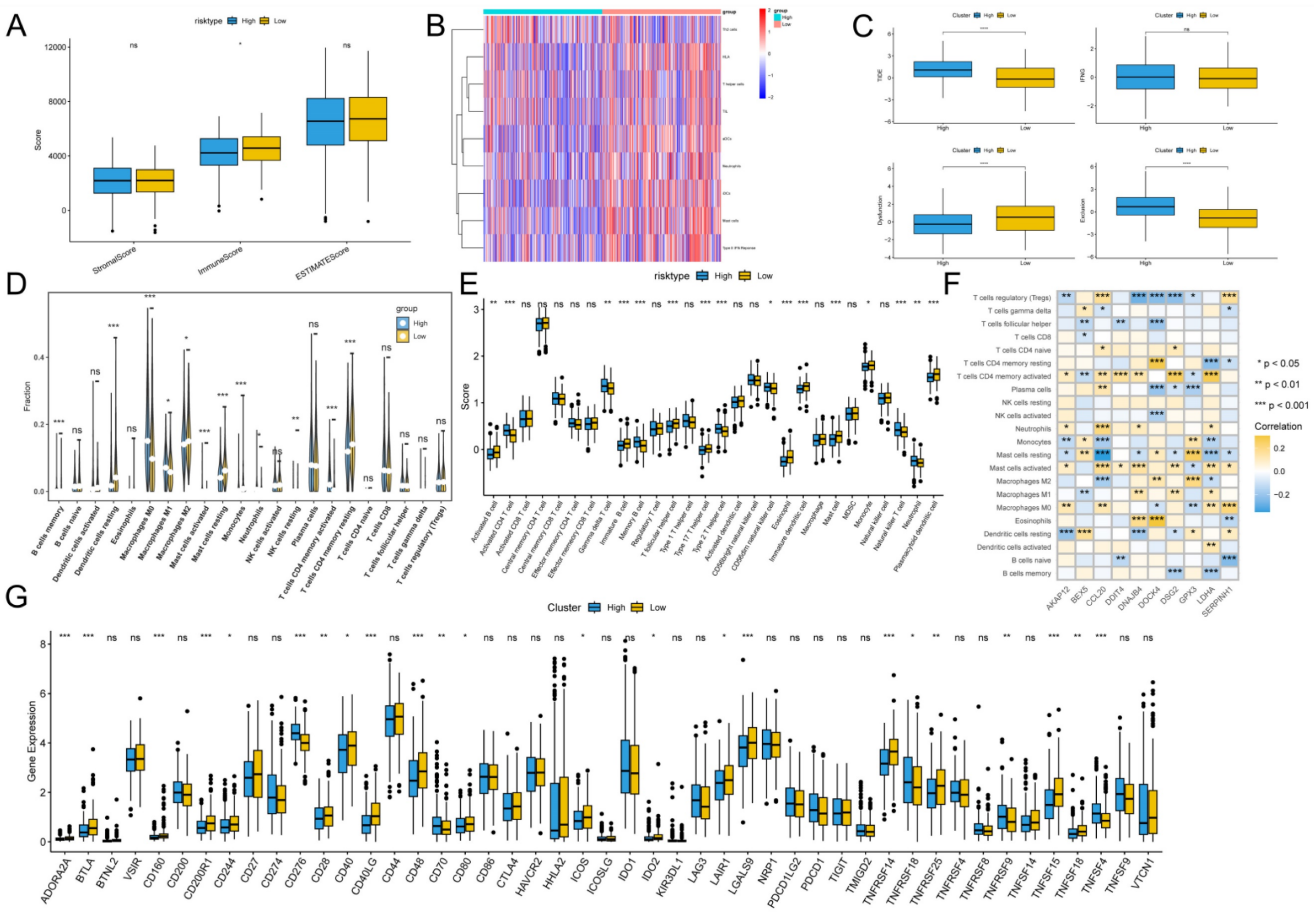
** Immunoscape and immunologic properties of G protein-coupled receptors.** A: Immunoscore, ESTIMATE score and stromal score were applied to determine the different immune status between the high and low risk groups; B: The activity of immune-related pathways was significantly different between the high risk and the significant differences between high and low risk groups. C: Immunotherapy escape status was assessed using TIDE in high and low risk groups; D, E: The abundance of infiltrating cell types between each risk group was quantified by the CIBESORT algorithm (D) and the ssGSEA algorithm (E) between the high and low risk groups. F: Correlation of immune infiltrating cells with the GPCRRS gene. G: The expression of immune checkpoints in high- and low-risk groups.

To assess the immune infiltration status of the LUAD samples, we used the ESTIMATE algorithm to calculate the immunization score, stromal score, and ESTIMATE score for the GPCRRS risk group. The results showed that the immune score was significantly higher in the low-risk group (**Figure 10A**). In addition, using the ssGSEA algorithm, immune-related pathway scores were obtained in this study. The low-risk group showed greater activity in HLA, TIL and Type II IFN Respone (**Figure 10B**). In addition, we estimated the TIDE score between the two groups using the TIDE online website and found that the TIDE score was significantly higher among the high-risk group, suggesting that the high-risk group may have stronger immunotherapy escape (**Figure 10C**). To further analyze the differences in specific immune cell infiltration between the high- and low-risk groups, we quantified the abundance of immune cell infiltration in each sample using the CIBERSORT algorithm (**Figure 10D**). We found that M0 macrophages and M1 macrophages were more abundant in the high-risk group. Whereas B cells Memory, Monocytes, T cells CD4 memory resting and mast cell resting, were more abundant in the low-risk group. Similar results were obtained by applying the ssGSEA algorithm for validation (**Figure 10E**). In addition, we found that 10 genes within GPCRRS were strongly correlated with immune scores and highly correlated with tumor-infiltrating immune cells, among which CCL20 was positively correlated with mast cells activated and T cells CD4 memory activated, and GPX3 and DOCK4 were positively correlated with M2 macrophages (**Figure 10F**). In addition, of the 47 immune checkpoint-associated genes obtained from the literature^41^ the expression of 23 checkpoint-associated genes (48.94%) differed significantly between molecular subtypes (**Figure 10G**). The expression of most of these genes was significantly higher in the Low group than in the High group, suggesting that patients of different subtypes may respond differently to immunotherapy.

### Relationship between G protein-coupled receptors and immunotherapy

**Figure 11:**
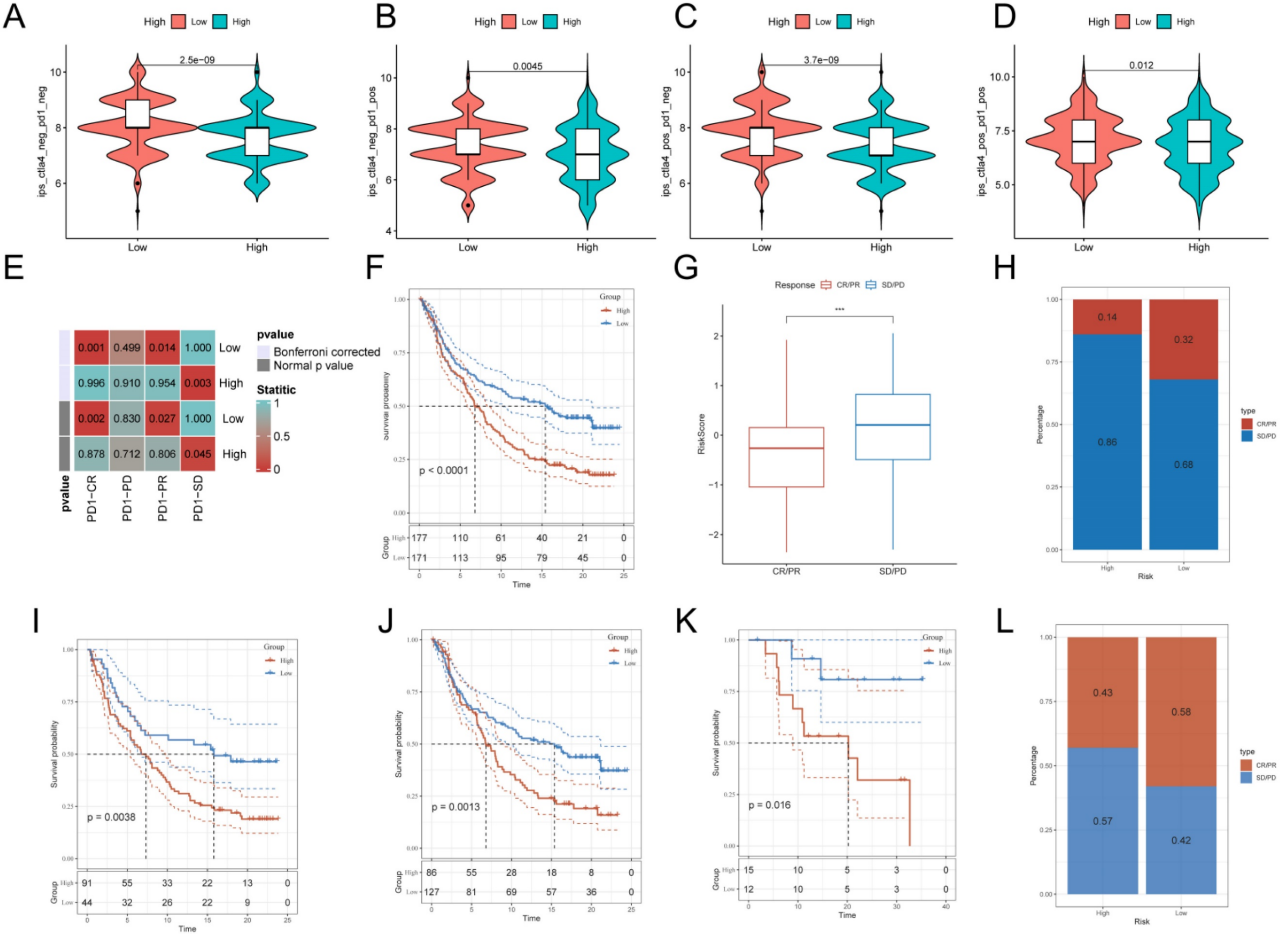
** Relationship between G protein-coupled receptors and immunotherapy.** A:CTLA4-negative/PD-1-negative;B:CTLA4-negative/PD-1-positive; C:CTLA4-positive/PD-1-negative; D: CTLA4-positive/ PD-1 positive; E: SubMap tool analysis showed that high and low risk groups predicted response to anti-PD-1 therapy. Obtained p-values were adjusted by the Bonferroni method. F: Prognostic differences between risk subgroups in the IMvigor210 cohort. G: Differences between immunotherapy responses based on risk scores in the IMvigor210 cohort. H: Distributions of immunotherapy responses based on risk subgroups in the IMvigor210 cohort. I: Distribution of immunotherapy responses based on early-stage (I-II) and early-stage (I-II) in the IMvigor210 cohort. Difference in prognosis between risk subgroups based on early stage (stage I-II) in the IMvigor210 cohort. J: Difference in prognosis between risk subgroups based on late-stage patients (stage III-IV) in the IMvigor210 cohort. K: Difference in prognosis between risk subgroups in the GSE78220 cohort. L: Distribution of immunotherapy responses based on risk subgroups in the GSE78220 cohort. (**P < 0.01; ***P < 0.001; ****P < 0.0001).

After assessing the immune infiltration, to further investigate the potential correlation between GPCRRS and immunotherapy, IPS was used to assess the therapeutic efficacy of immune checkpoint inhibitors, which showed that the efficacy of the low-risk group was significantly better than that of the high-risk group, regardless of the status of CTLA-4 and PD-1 (**Figure 11A-D**). We performed SubMap analysis to assess the anti-PD-1 immunotherapy response in the high- and low-risk group immunotherapy. The results showed that the low-risk group predicted partial and complete response (PR/CR) to anti-PD-1 immunotherapy, while the high-risk group predicted resistance (SD) to anti-PD-1 immunotherapy (**Figure 11E**). In addition, we analyzed the response to PD-L1 blockade immunotherapy in the IMvigor210 and GSE78220 cohorts. 348 patients in the IMvigor210 cohort showed different responses to anti-PD-L1 receptor blockers, including stable disease (SD), partial remission (PR), complete remission (CR) and disease progression (PD). We found that CR/PR patients had lower risk scores than SD/PD patients (**Figure 11G**). In addition, the proportion of SD/PD patients was lower in the low-risk group than in the high-risk group (**Figure 11H**). Our analysis of the IMvigor210 cohort showed that patients in the low-risk group had significantly better clinical outcomes than patients in the high-risk group (**Figure 11F**). In addition, we found significant survival differences between different risk groups not only in stage I+II patients but also in stage III+IV patients (**Figure 11I, J**). To confirm our findings, we included the GSE78220 cohort in further analysis. In contrast to the results obtained in IMvigor210, the proportion of PR/CR patients was lower in the high-risk group and good survival significance was observed in the low-risk group (**Figure 11K-L**). These results suggest that GPCRRS can assess the efficacy of immunotherapy and that patients with lower risk scores may better benefit from immunotherapy.

### Identification of key regulators of GPCRRS

**Figure 12:**
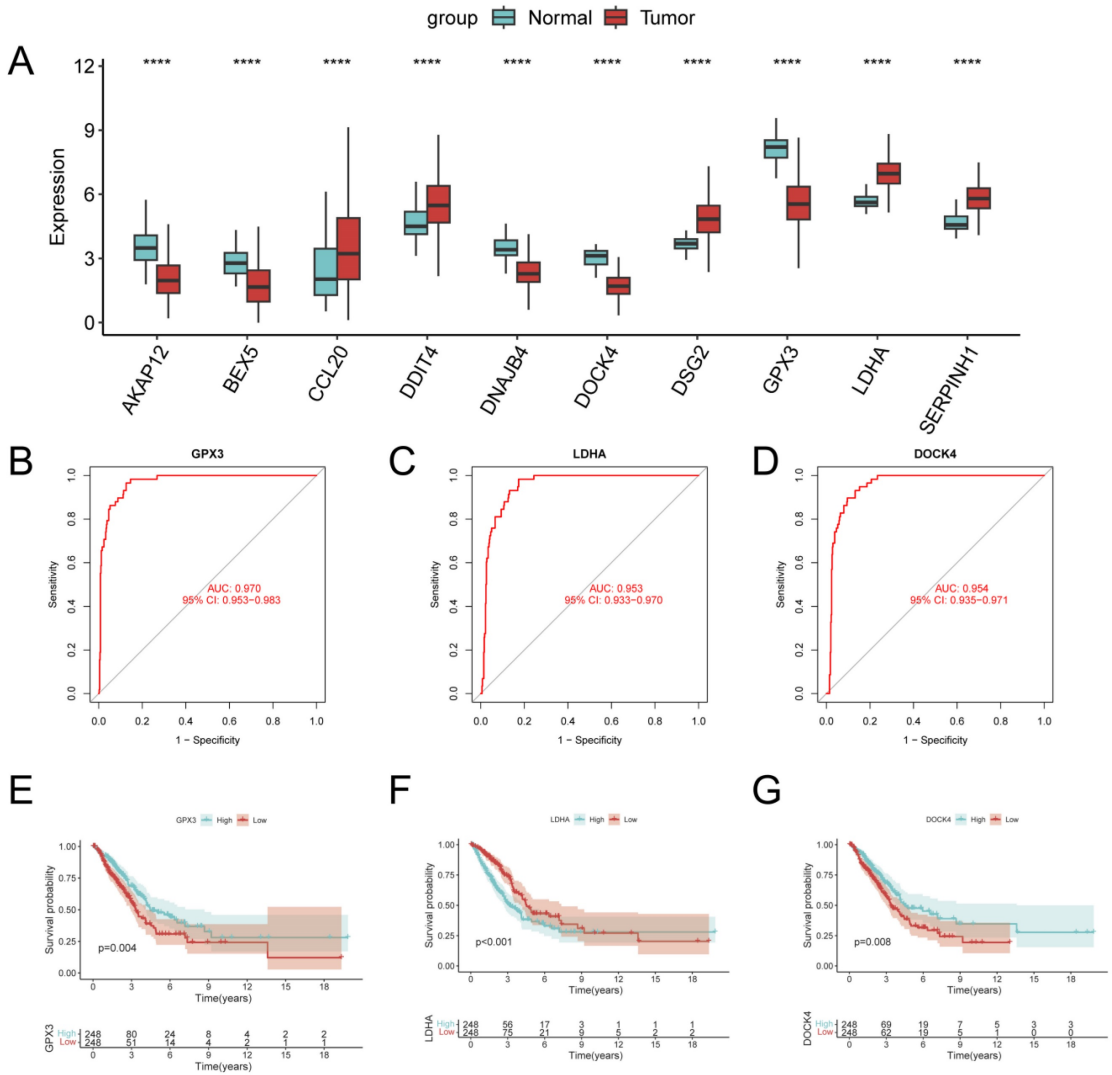
** Identification of key regulators of GPCRRS.** A: Expression of GPCRRS gene in cancer and paracancer; B: ROC diagnostic curves of GPX3; C: ROC diagnostic curves of LDHA; D: ROC diagnostic curves of DOCK4; E: Survival curves of GPX3; F: LDHA survival curve; G: survival curve of DOCK4. (**P < 0.01; ***P < 0.001; ****P < 0.0001).

In order to identify the key regulators in the risk subgroups, first we verified the mRNA expression levels of these 10 genes, and found that CCL20, DDIT4, DSG2, LDHA, and SERPINH1 were highly expressed in tumors, whereas GPX3, BEX5, and DOCK4 were highly expressed in normal tissues (**Figure 12A**). In addition, we used ROC diagnostic curves to screen for key regulators, and we found that the only ones with ROC>0.95 were GPX3, LDHA, and DOCK4, and thus we considered these three genes to be key regulators of GPCRRS (**Figure 12B-D, Supplementary Figure 2A-G**). We plotted KM curves to verify the survival of these three key genes, and we found that high expression of GPX3 and DOCK4 had a good prognosis, while low expression of LDHA had a good prognosis (**Figure 12E-G, Supplementary Figure 3A-G**). The results showed that GPX3, DOCK4, and LDHA were the key regulators of GPCRRS.

### Analysis of the correlation between the GPCRRS and drug sensitivity and validation of gene expression

**Figure 13:**
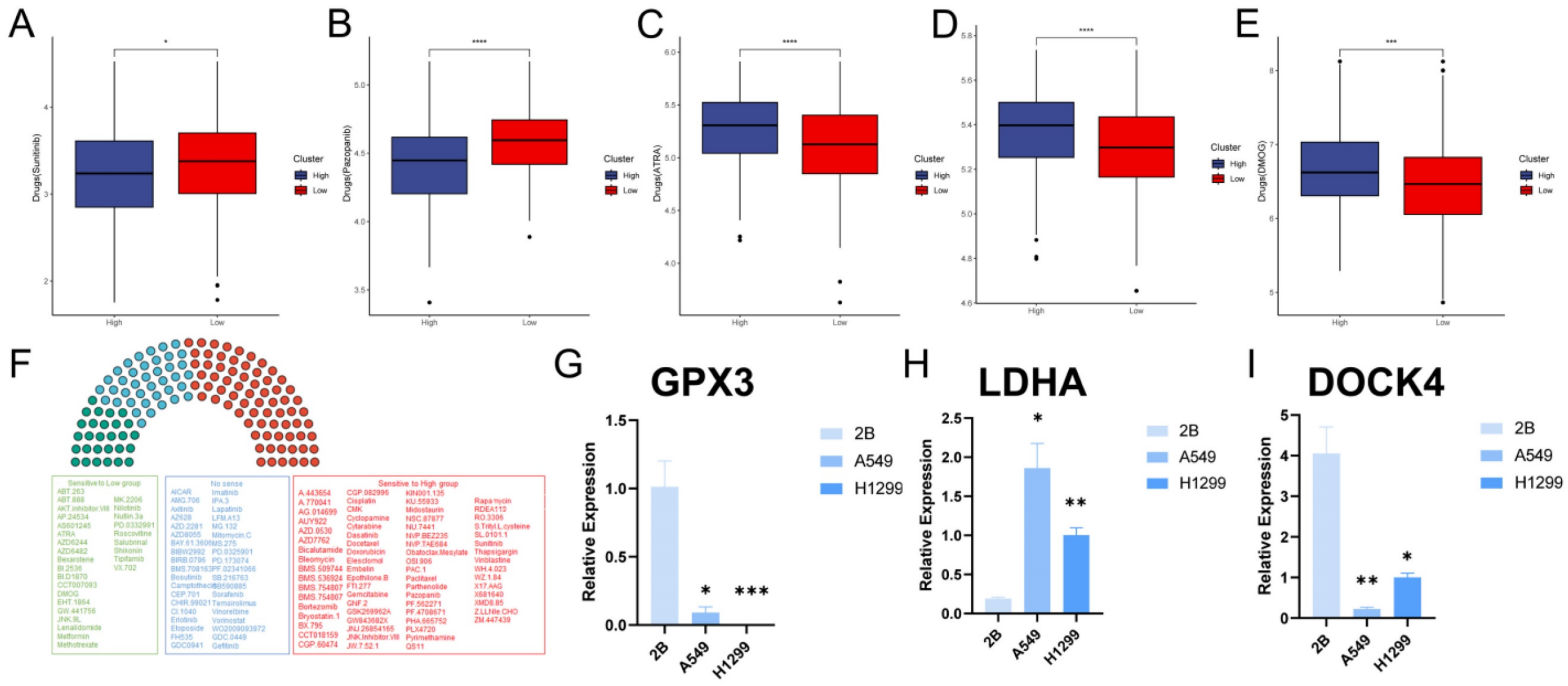
** Between GPCRRS and drug sensitivity regarding and validation of key genes.** A-E: Comparison of GPCRRS high and low risk groups on drug sensitivity F: Analysis of high and low risk group population on drug sensitivity. Green, sensitivity to low-risk scoring patients; red, sensitivity to high-risk patients; blue, no significance; G-I: Validation of GPX3 (G), LDHA (H), and DOCK4 (I) expression by RT-qPCR in normal cell line (2B) and two LUAD cell lines (H1299 and A549). (*P < 0.05; **P < 0.01; ***P < 0.001; ****P < 0.0001).

Over the past few decades, significant progress has been made in exploring the molecular mechanisms of LUAD progression, leading to the development of precision therapeutics such as tyrosine kinase inhibitors (TKIs)^42^. With the advent of molecular profiling, it has become clear that lung adenocarcinoma is a genetically heterogeneous disease characterized by a series of driver mutations and alterations suitable for targeted therapy^43^. However, drug resistance is a common problem due to the highly dynamic and heterogeneous tumor microenvironment^44^, ^45^. To this end, we examined the sensitivity of GPCRRS risk subgroups to two tyrosine kinase inhibitors and found that sunitinib and pazopanib were significantly elevated in the low-risk group (**Figure 13A, B**), as well as the other drugs, ATRA, ABT.888, and DMOG, were significantly elevated in the high-risk group (**Figure 13C-E**), and the risk scores were differently correlated to the drugs (**Supplementary Figure 4A-E**). These results suggested that patients in the high-risk group responded better to sunitinib or pazopanib treatment, whereas patients in the low-risk group might be more sensitive to ATRA, ABT.888 and DMOG. In addition we further performed drug sensitivity analysis to predict the IC50 of 139 chemotherapeutic agents (**Figure 13F**). The results showed that 72 drugs in the high-risk group had low IC50 values, suggesting sensitivity. Patients in the low-risk group were sensitive to 28 drugs. In conclusion, these results provide a reference standard for therapeutic analysis of LUAD patients.

Finally, we evaluated the expression of the three core genes in GPCRRS in three cell lines, including one normal cell line (2B) and three lung adenocarcinoma cell lines (A549 and H1299) (**Figure 13G-I**). The results showed that GPX3 and DOCK4 expression was significantly upregulated in normal cell lines, while LDHA expression was significantly upregulated in tumor cell lines.

## Discussion

Lung cancer is the most common pathologic type, accounting for nearly 40% of all lung cancer subtypes, and is characterized by rapid progression, severe prognosis, and early relapse^46^. The prognosis of lung cancer is severe and early recurrence. Due to tumor heterogeneity and adverse events, immunotherapy has become an important therapeutic strategy for low response rates^47^, ^48^. The Identification of effective biomarkers is the key to improving the efficacy of immunotherapy. Currently, a variety of biomarkers are used to assess the response to immunotherapy, including tumor mutation burden^49^, PD-1, PD-L1, CTLA-4^50^, TIGIT^51^, ^52^, and neoantigens^53^. However, the overall survival of patients with LUAD remains suboptimal, with a 5-year survival rate of 19%^3^. In addition, even in early-stage LUAD patients, the recurrence rate remains at 30-45% within 5 years after surgery^54^, ^55^. The development of new biomarkers for LUAD Therefore, the development of new biomarkers, therapeutic targets, and drugs is essential to improve the early diagnosis and outcome of LUAD patients with the aim of improving their survival.

In recent years, several studies have aimed to establish a gene signature based on programmed cell death to better understand the prognostic classification of LUAD. For example, Wang Z et al.^56^ reported a metabolism-related prognostic signature that predicted overall survival in LUAD. Similarly, Shi R et al.^57^ developed a prognostic signature based on hypoxia-derived related genes to predict prognosis in LUAD. Gao J et al.^58^ constructed a model related to autophagy in radiotherapy that performed well in predicting overall survival in LUAD. As well as Xu F et al.^59^ constructed a prognostic model based on m6A-associated lncRNAs to validate the prognosis of LUAD, and all of these studies showed a certain degree of predictive ability for the prognosis of LUAD patients, immune response, etc. G protein-coupled receptors are closely associated with tumorigenesis and cancer survival. However, only a few studies have focused on G protein-coupled receptor-related gene-based models.

Wang et al. screened for G protein-coupled receptor-related genes through immune infiltration-related genes^60^; Liu et al. studied the effects of G protein-coupled receptor antagonists on lung adenocarcinoma 61; Jala VR, Li ZH, Wu G and Yao S et al. studied single gene of G protein coupled receptor^62^-^65^; Touge H et al. study was the effect of G protein coupled receptor on the morphology of lung adenocarcinoma cells^66^; Khan M's review type of study mainly talked about the relevance of GPCR in the treatment of lung adenocarcinoma^67^; Gao Y and Fujimoto J was mainly experimental and did not use bioinformatics on a large scale^68^, ^69^.Our study is G protein-coupled receptor fully characterised and some of the genes have not been studied yet.

We obtained G protein-coupled receptor related genes from single cell transcriptomes using scRNA-seq data from the GSE149655. The TCGA-LUAD data and the GSVA algorithm were then used to identify the key modules most relevant to the progression of G protein-coupled receptor, and differential genes were obtained by differential analysis of the TCGA-LUAD data. When we selected the intersection of G protein-coupled receptor marker genes and differential genes, we finally found 66 genes involved in G protein-coupled receptor both in the single-cell transcriptome and in the bulk transcriptome. We then built a new prognostic model using a combination of machine learning algorithms to screen for 10 genes. Second, we modeled LUAD patients using GPCRRS to analyze high and low patient responses to immunotherapy and sensitivity to first-line drugs, including tyrosine kinase inhibitors (TKIs). In addition, unlike previous studies, we performed a comprehensive multi-omics analysis including genome, single-cell transcriptome, and overall transcriptome to gain a deeper understanding of GPCRRS. Significant prognostic differences were found between the two groups, demonstrating the independent predictive value of the GPCR profile we created for LUAD.ROC curve and calibration curve analyses demonstrated the superior predictive efficacy of the GPCR profile for patient prognosis. In addition, the line graphs we created demonstrate in a promising way the superiority of the GPCR signature relative to other clinically used indications.

In this study, a new computational framework is used to identify stable and robust prognostic features, GPCRRS. the framework contains 10 machine learning algorithms and their multiple combinations^70^. Using this framework, we reduced the dimensionality of the variables, simplified the model, and successfully fitted a consensus model with high predictive accuracy and translatability. Through KM curve analysis and multivariate analysis, we determined that the GPCRRS could stratify the risk of LUAD patients according to OS, PFS, and DSS, as well as serve as an independent prognostic factor for these outcomes. In addition, the predictive accuracy of the GPCRRS was significantly better than other clinical characteristics. The stability of the prognostic stratification of clinical subgroups reaffirmed the robustness of the GPCRRS. In addition, we observed that GPCRRS was associated with high grading and late stage of LUAD, which correlates with poor clinical outcomes.

Notably, our study demonstrated that GPCRRS not only predicts the prognosis of LUAD patients, but also has the potential to predict the development of LUAD. GPCRRS showed good diagnostic performance on the TCGA, GSE31210, and GSE50081 datasets. The AUCs of the prognostic models constructed by Wu et al. by selecting the ICD-related DAMP gene were 0.73,0.68 and 0.67^71^; the AUCs of the prognostic models constructed by Jiang et al. through the single-cell transcriptome and bulk transcriptome were 0.669,0.674 and 0.642^72^; the AUCs of the prognostic models constructed by Yang et al. through the lncRNAs-related immune gene constructed prognostic models with AUCs of 0.727,0.709 and 0.675, respectively^73^. Our AUC results were 0.758, 0.740, and 0.700. These results suggest that GPCRRS has good predictive ability to predict the prognosis of LUAD. Therefore, risk stratification based on GPCRRS can be used to identify individuals at high risk for LUAD. For these patients, clinicians can develop interventions such as regular physical examinations, lifestyle interventions, and specific preventive medications.

To provide a convenient tool to quantify survival in LUAD, we constructed a nomogram survival plot combining GPCRRS and clinical features. the nomogram ROC curve was highly accurate in prediction, and the ROC curve and C-index were shown to have good discriminatory properties. The calibration curves further confirmed the accuracy of the nomogram by showing close agreement between predicted and observed survival. Importantly, the application of the nomogram resulted in a greater net benefit compared with other clinical features, suggesting that the nomogram has great potential as a promising and convenient clinical tool for predicting survival in patients with LUAD.

G-protein-coupled receptors (GPCRs) are the largest family of cell surface signaling receptors known to play important roles in a variety of physiological functions, including tumor growth and metastasis^8^. Thus G protein-coupled receptors may be closely related to tumor immunotherapy and the immune landscape. We wanted to gain a deeper understanding of the immune microenvironment associated with GPCRs in LUAD and their role in immunotherapy. To accomplish our goal, we used several algorithms including ESTIMATE, CIBERSORT, ssGSEA, and others. These algorithms allowed us to identify immune cell infiltration in LUAD. Our results showed that the low-risk group had a highly infiltrated immune microenvironment, in contrast to the high-risk group, which had a less infiltrated immune microenvironment. In addition, we assessed the immunotherapy response and drug sensitivity between the two groups by algorithms such as TIDE, which provides some basis for clinical treatment. We further validated the ability of GPCRRS to predict immunotherapy response using the real cohort SubMap and IMvigor210. Notably, the proportion of complete remission/partial remission (CR/PR) patients was significantly higher in the low-risk group. In addition, CR/PR patients had lower risk scores than stable disease/progressive disease (SD/PD) patients. These findings confirm the ability of GPCRRS to predict response to immunotherapy and suggest that low-risk populations may derive greater benefit from immunotherapy. Our findings suggest that GPCRRS may serve as a valuable biomarker for immunotherapy in patients with LUAD.GPCRRS can be used to identify which patients with LUAD may benefit from immunotherapy prior to treatment initiation.

To evaluate the predictive value of GPCRRS in determining the prognosis of LUAD, we initially selected 10 GPCRs to build a risk score model to predict the overall survival of LUAD patients. Later, we found that among these 10 genes, 3 genes had very good diagnostic efficacy, so we thought that these three genes might be the key regulators of GPCRRS. Interestingly, we found that previous studies reported that these genes play an important role in cancer progression: the LDHA activates GTPase Rac1 in a manner independent of its glycolytic enzyme activity thereby promoting cancer progression^74^; DOCK4 is a key component of the TGF-b/Smad pathway and promotes lung ADC cell extravasation and metastasis^75^; GPX3 is a potent tumor development suppressor^76^. Although the regulatory roles of these GPCRs have been studied in various cancers, few researchers have systematically evaluated their prognostic value in LUAD. Overall, we were the first to investigate the prognostic significance of "G protein-coupled receptors" in patients with LUAD and pioneered the development of a risk scoring model based on G protein-coupled receptor-related genes.

However, this study still has some limitations, firstly we used samples from public databases and analyzed them, so inherent cases may affect the results. More convincing studies are needed to confirm our findings. Second, although we evaluated and validated the GPCRRS model in the training set and external validation set, large-scale, multicenter prospective studies are needed to further confirm our findings. More *in vitro* and *in vivo* studies are needed to elucidate the biological functions of GPCRs-related genes in LUAD. In conclusion, although we predicted that the expression and prognostic role of genes in GPCRRS at the protein level deserve further study. Future studies must explore the potential mechanisms between GPCRRS gene expression and LUAD prognosis.

In conclusion, a G protein-coupled receptor-related gene-based signature was identified and validated to have strong properties to predict prognosis and immunotherapy response in LUAD patients. It can be used as a prognostic biomarker for individualized predictive clinical decision-making and helps to select appropriate patients who can benefit from immunotherapy.

## Supplementary Material

Supplementary figures.

## Figures and Tables

**Table 1:** Correlation between high and low risk groups and clinicopathologic characteristics of lung adenocarcinoma patients.

Characteristics	High (N=248)	Low (N=248)	P-value
Age			
<=65	123 (49.6%)	113 (45.6%)	0.414
>65	120 (48.4%)	130 (52.4%)	
Unknown	5 (2.0%)	5 (2.0%)	
Gender			
Male	127 (51.2%)	140 (56.5%)	0.28
Female	121 (48.8%)	108 (43.5%)	
Stage			
I	109 (44.0%)	157 (63.3%)	5.6e-05
II	70 (28.2%)	48 (19.4%)	
III	53 (21.4%)	26 (10.5%)	
IV	14 (5.6%)	11 (4.4%)	
Unknown	2 (0.8%)	6 (2.4%)	
T stage			
T1	64 (25.8%)	101 (40.7%)	0.003
T2	144 (58.1%)	122 (49.2%)	
T3	28 (11.3%)	16 (6.5%)	
T4	11 (4.4%)	7 (2.8%)	
Unknown	1 (0.4%)	2 (0.8%)	
N stage			
N0	140 (56.5%)	181 (73.0%)	0.00045
N1	57 (23.0%)	37 (14.9%)	
N2	46 (18.5%)	22 (8.9%)	
N3	1 (0.4%)	1 (0.4%)	
Unknown	4 (1.6%)	7 (2.8%)	
M stage			
M0	167 (67.3%)	162 (65.3%)	0.613
M1	14 (5.6%)	10 (4.0%)	
Unknown	67 (27.0%)	76 (30.6%)	
