# Supplementary material for: Multi-omics identification of GPCR gene features in lung adenocarcinoma based on multiple machine learning combinations
Source: J Cancer. 2024 Jan 1;15(3):776–95. doi: 10.7150/jca.90990 (PMC10777041; doi:10.7150/jca.90990)
Supplement: Supplementary file 1 — Supplementary figures. [file jcav15p0776s1.pdf]

Supplementary Figures:

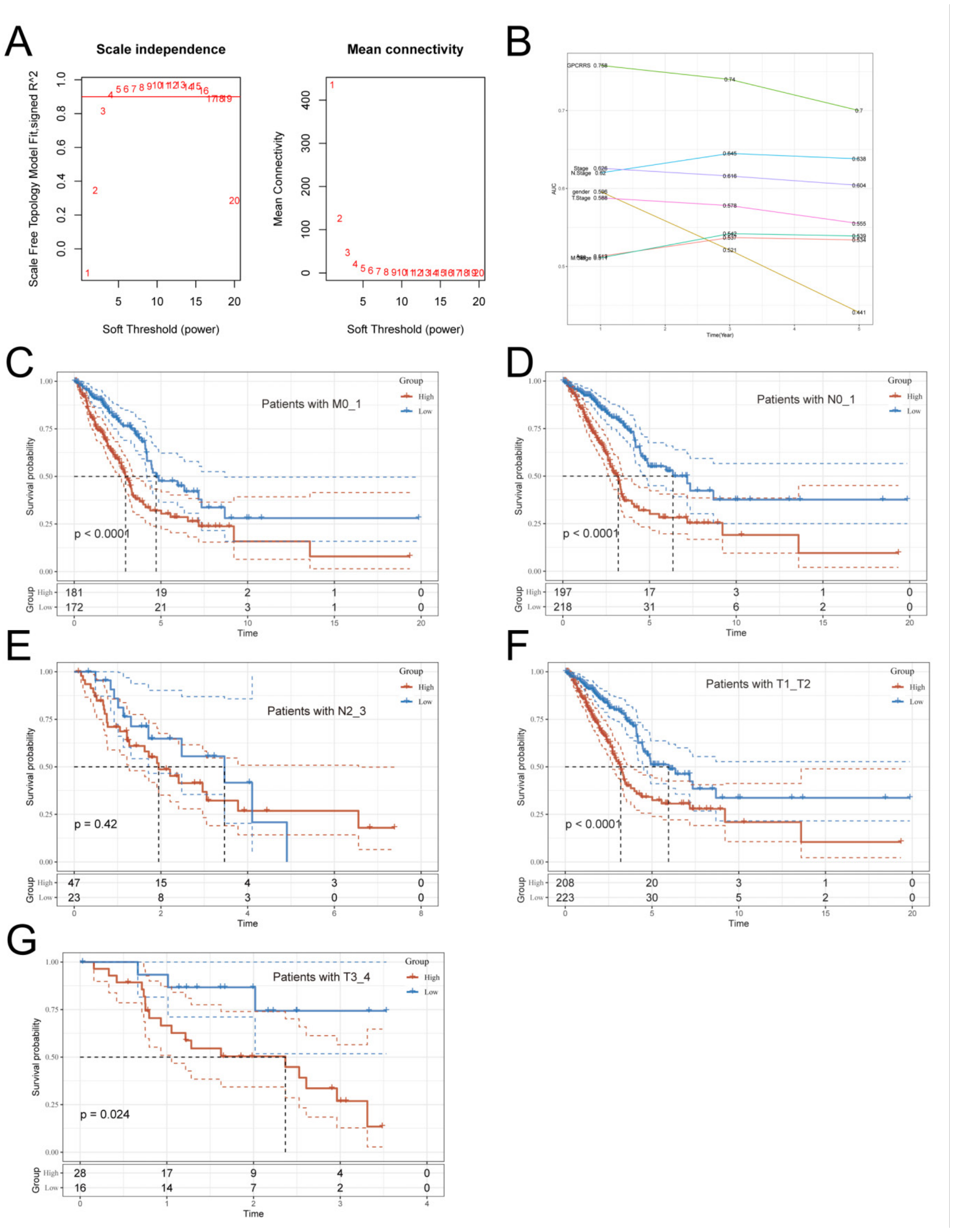

Supplementary Figure.1

Identification of soft queer values and KM curves for clinical features of GPCRRS.

(A)WGCNA identifies soft queues;（B)Comparison of GPCRRS with other clinical features in the TCGA training set;（C-G）KM curves demonstrating the stability of each clinical feature of GPCRRS, including M-stage(C), N-stage（D,E）, and T-stage(G).

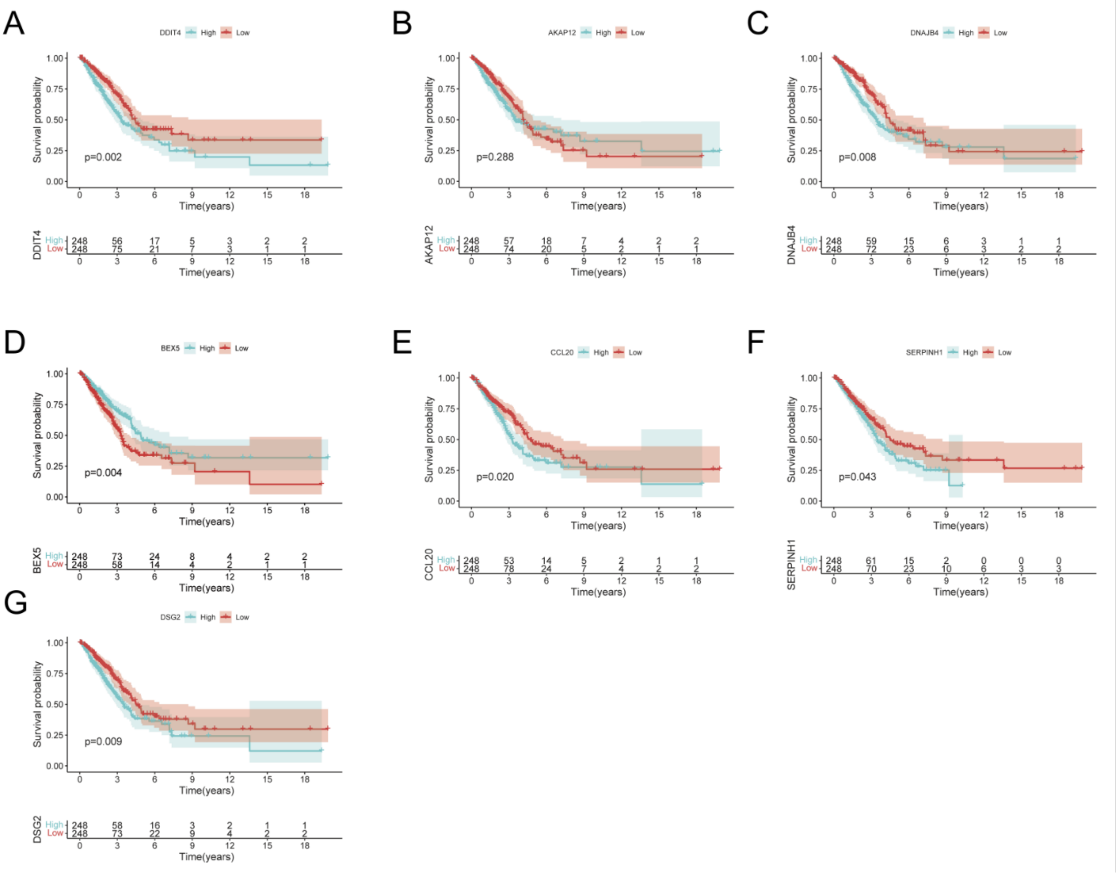

Supplementary Figure.2

Prognosis of the GPCRRS gene.

KM curves demonstrating the prognostic relationship of GPCRRS genes in high and low expression groups including DDIT4(A),AKAP12(B),DNAJB4(C),BEX5(D),CCL20(E),SERPINH1(F),DSG2(G).

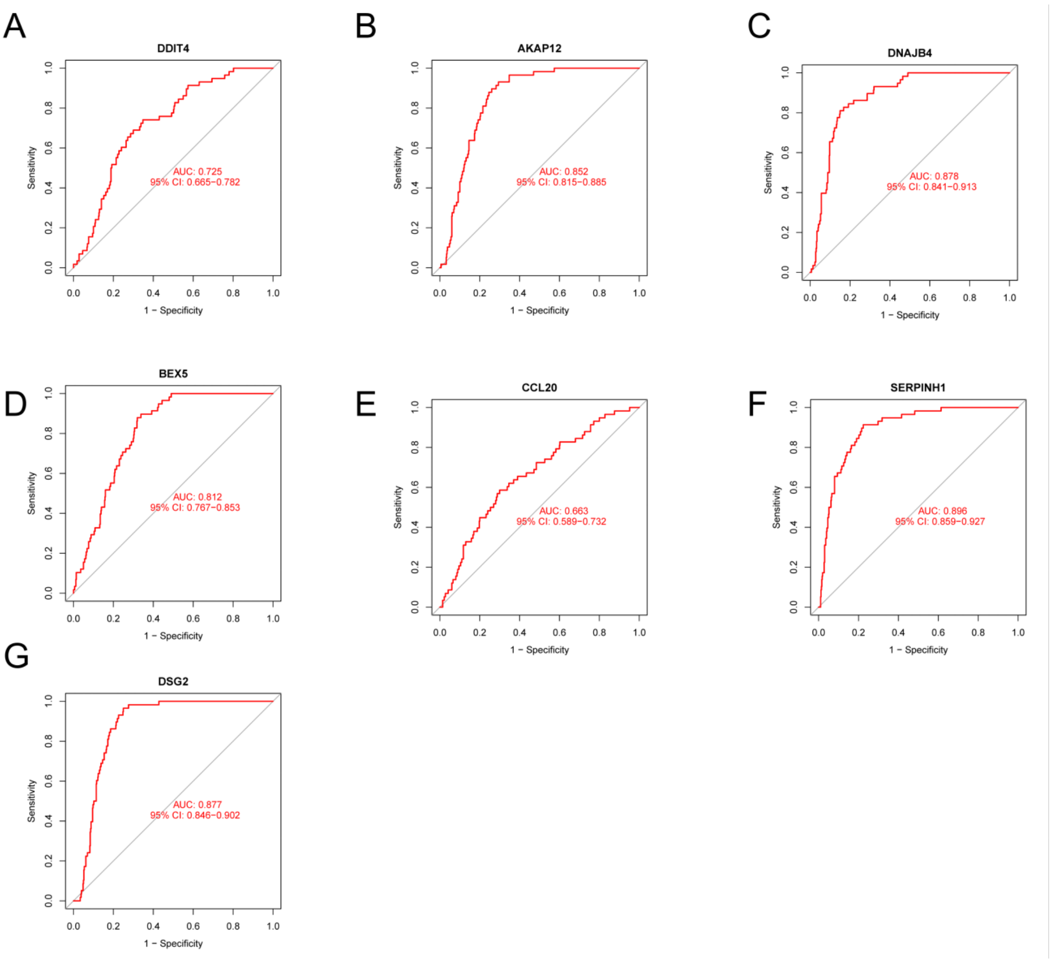

Supplementary Figure.3

ROC diagnostic curve;

ROC diagnostic curve for the GPCRRS gene including DDIT4(A),AKAP12(B),DNAJB4(C),BEX5(D),CCL20(E),SERPINH1(F),DSG2(G).

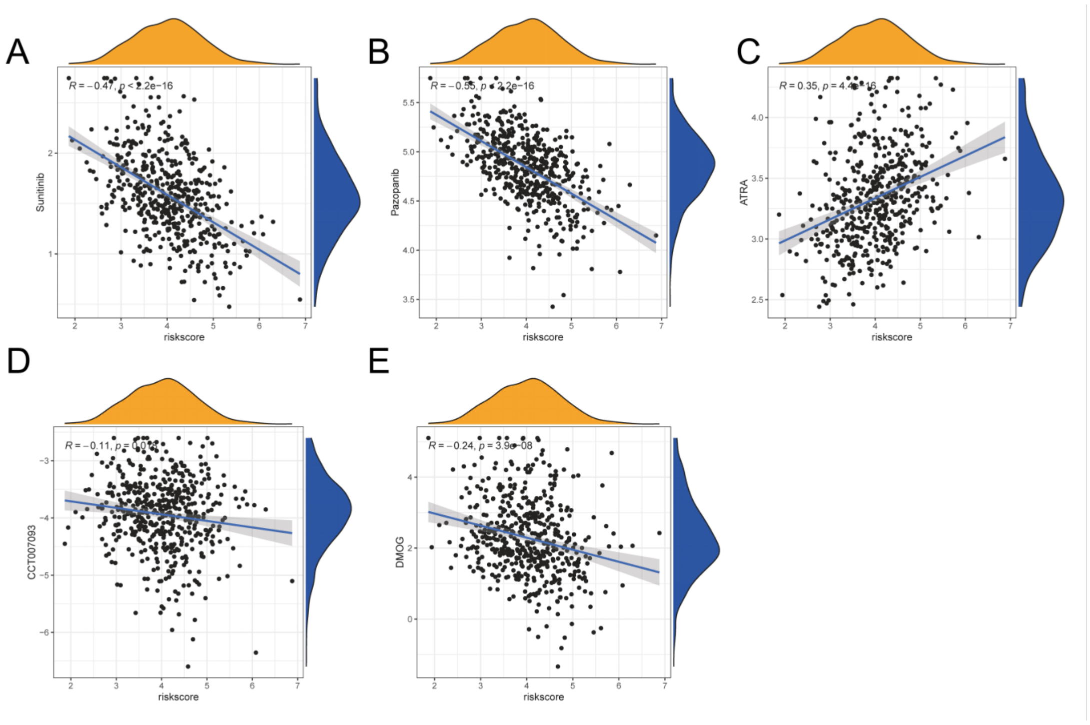

Supplementary Figure.4

Relevance of drugs;

Correlation between GPCRRS and drugs including Sunitinib(A), Pazopanib(B), ATRA(C), CCT007093(D), DMOG(E).
